# How Simple Hypothetical-Choice Experiments Can Be Utilized to Learn Humans’ Navigational Escape Decisions in Emergencies

**DOI:** 10.1371/journal.pone.0166908

**Published:** 2016-11-21

**Authors:** Milad Haghani, Majid Sarvi, Zahra Shahhoseini, Maik Boltes

**Affiliations:** 1 Centre for Disaster Management and Public Safety, School of Engineering, The University of Melbourne, Stationary Ln, Parkville VIC 3052, Australia; 2 Jülich Supercomputing Centre, Forschungszentrum Jülich, 52425 Jülich, Germany; Nanjing University, CHINA

## Abstract

How humans resolve non-trivial tradeoffs in their navigational choices between the social interactions (e.g., the presence and movements of others) and the physical factors (e.g., spatial distances, route visibility) when escaping from threats in crowded confined spaces? The answer to this question has major implications for the planning of evacuations and the safety of mass gatherings as well as the design of built environments. Due to the challenges of collecting behavioral data from naturally-occurring evacuation settings, laboratory-based virtual-evacuation experiments have been practiced in a number of studies. This class of experiments faces the traditional question of contextual bias and generalizability: How reliably can we infer humans’ behavior from decisions made in hypothetical settings? Here, we address these questions by making a novel link between two different forms of empirical observations. We conduct hypothetical emergency exit-choice experiments framed as simple pictures, and then mimic those hypothetical scenarios in more realistic fashions through staging mock evacuation trials with actual crowds. Econometric choice models are estimated based on the observations made in both experimental contexts. The models are contrasted with each other from a number of perspectives including their predictions as well as the sign, magnitude, statistical significance, person-to-person variations (reflecting individuals’ perception/preference differences) and the scale (reflecting context-dependent decision randomness) of their inferred parameters. Results reveal a surprising degree of resemblance between the models derived from the two contexts. Most strikingly, they produce fairly similar prediction probabilities whose differences average less than 10%. There is also unexpected consensus between the inferences derived from both experimental sources on many aspects of people’s behavior notably in terms of the perception of social interactions. Results show that we could have elicited peoples’ escape strategies with fair precision without observing them in action (i.e., simply by using only hypothetical-choice data as an inexpensive, practical and non-invasive experimental technique in this context). As a broader application, this offers promising evidence as to the potential applicability of the hypothetical-decision experiments to other decision contexts (at least for non-financial decisions) when field or real-world data is prohibitively unavailable. As a practical application, the behavioral insights inferred from our observations (reflected in the estimated parameters) can improve how accurately we predict the movement patterns of human crowds in emergency scenarios arisen in complex spaces. Fully-generic-in-parameters, our proposed models can even be directly introduced to a broad range of crowd simulation software to replicate navigation decision making of evacuees.

## Introduction

Over the last two decades, a rapidly growing interest has developed among researchers in a variety of scientific disciplines towards understanding the nature of pedestrian crowds. A great deal of research has been carried out to enhance the state of knowledge on behavioral mechanisms that govern the motion of large numbers of pedestrian humans in different environments and settings. Attention to the problem was particularly prompted by the occurrence of a number of recent mass crowd events culminated with disasters and casualties [1, 2] that heightened awareness as to the importance of crowd management and control. Owing to the growing urban populations and the increasing frequency of similar mass events, this continues to be a critical safety-related research stream [3, 4]. It essentially aims at developing reliable forecast tools that enable planners to reduce the likelihood of such disasters by conducting virtual (or simulated) evaluations on the design and performance of public crowded facilities. Despite major advances in this field, it is still thought of as an intractable problem many fundamental aspects of which are yet to be investigated [5]. The intrinsic complexity and variability of human behavior (as the constituent elements of crowds), the variety of circumstances and geometric features that need to be dealt with (ranging from large open areas, to complex confined spaces such as public transportation facilities or high-rise buildings), and the variety of contexts (such as entertainment events, sport events, political protests; or unanticipated emergency situations) that can give rise to different forms of behaviors in a crowd are among the primary challenges involved [6]. However, it is believed that the key challenge arises from the sparseness (or often lack) of the reliable empirical data [3].

Former studies have looked at the problem from a wide range of perspectives such as certain macro-scale phenomena that emerge as a result of the collective motion of crowds [7], issues affecting the psychological state of individuals in large crowds [8, 9], or the impact of individuals’ social association within a crowd on their behavior [10, 11]. One particular topic to which a great deal of attention has been paid is emergency evacuations of crowds from confined environments.

Introduction of the “panic escape” model proposed by Helbing et al. [12] inspired by the notion of Newtonian mechanics was followed by the development of a great number of models and methodological approaches in the field. One of the main features of this model is the recognition of individual escapees as the main elements of crowds, as opposed to more traditional approaches that assume escaping crowds as continuous fluid-type bodies [13, 14]. This individual-centered approach, in general, postulates that the phenomena that emerge at aggregate level are engendered as a result of the disaggregate-level interactions. Owing to the reasonableness of this assumption, the approach has appealed to many researchers in this field. However, it requires replication and modeling of the relevant decisions made by each individual evacuee (as modeling entities).

Two primary types of decisions pertain to the problem, one relating to the local (also known as “operational-level” or “walking”) decisions that individuals adopt to avoid collision with obstacles and other evacuees while progressing towards their target. The other type of decision basically relates to the rules and mechanisms that govern evacuees’ “global navigation” (i.e. choosing their target, such as the exit or route of navigation). There has been an awful lot of effort towards the former type of decisions in the modern literature. A number of data provision approaches and methods have been employed ranging from experiments with stressed insects (typically, ants) [15–21] or mice [15, 22, 23] to controlled laboratory experiments [24–26] and video-analysis of pedestrian movements in naturally-occurring environments (although not necessarily during evacuations) [27, 28]. Also, a number of influential theoretical works have proposed adequately-defined modeling frameworks validated or calibrated based upon empirical observations [29, 30]. In contrast however, far less is known about the global navigation behavior of evacuees such as their exit or route choice from a modeling point of view.

With regard to knowing how evacuees make their global navigation decisions, fundamental behavioral questions are yet to be addressed as to (1) the factor(s) that contribute to exit decisions [5], (2) the way tradeoffs are made between them [31]; and (3) whether those factors and their influences dramatically differ from person to person [32]. The insufficiency of the behavioral data has thus far hindered making solid inferences from the existing literature to answer these questions. The nature of this type of decisions immediately rules out the relevance and possibility of employing certain data collection techniques, such as experiments with mass bodies of panicked insects or animals [33, 34], and leaves researchers with fewer options.

Researchers in this field have generally adopted two main approaches to gather empirical evidence for this particular problem. One has been conducting decision experiments in different forms of laboratory-type virtual-reality trials (i.e. virtual evacuations) ranging from static experiments in relatively simple geometric settings [35–37] to interactive experiments in the form of computer games [31, 38, 39]. A second approach that has been used by a fewer number of studies has been studying the behavior in action by conducting simplistic controlled mock evacuation trials in the “field” [40–42] or more precisely what experimental economists often refer to as “lab-like field experiments” or “framed field experiments” [43] (also, see [44] for a detailed taxonomy of the range of field experiments from an experimental economics perspective).

As mentioned earlier, modelling observations of emergency evacuations in fully-natural settings (i.e. field data in its classical definition and meaning) are extremely rare. Merely, for the sake of the easiness of the communication, the term “field-type” in this paper is used with a slightly twisted (although not awfully imprecise) meaning compared to its traditional concept. Here, we refer to the controlled simulated evacuation trials (that, strictly speaking, are still of laboratory nature but are more realistic than the purely hypothetical settings) as “field-type experiments”. This is purely to distinguish them from the counterpart experiments where the tight control of the experimenters is exerted over the design of the hypothetical decision situations (i.e. without any real action being observed) which we refer to as “hypothetical-decision laboratory experiments”.

To our knowledge, none of the studies that have made use of such field-type observations (i.e. controlled mock (or simulated) evacuation trials where the behavior of participants is observed in action) in the domain of exit decisions has provided choice data at disaggregate level that can serve as modeling materials. That has offered the experimenters of those studies not many analysis options beyond extracting simple descriptive statistics on macro-scale measures (i.e. total evacuation time, density distribution etc.)

Similar to many other areas of social sciences, notably economics, virtual environments in this context are believed to offer more flexibility at lower operational and logistics costs than those of the field-type experiments. Most importantly, they allow a perfect control of variation, often described as the “foundation of empirical scientific knowledge” [45] (see page 535). Particular to this context, they allow the analyst to take tight control of the moments and situations at which participants’ decisions are made, thus avoiding any ambiguity in terms of the set of alternatives or the level of attributes of those alternatives. Also the also the designer to stretch the attributes of alternatives to extreme levels, avoid correlations between attributes; and have participants make meaningfully complex tradeoffs [46]. Fairly large samples can be collected at reasonable amount of cost and within a reasonable course of time. More importantly, pertinent to this particular context again, they provide a safe and ethical environment for studying a range of potentially-dangerous situations that do not occur on a day-to-day basis in real life but might have significant consequences.

However, it is highly unknown whether or not the inferences made based on the hypothetical-choice experiments carried out in virtual settings are consistent with activities in natural environments and thus can reliably be generalized to real world situations. The problem is also referred to as “hypothetical bias” or “contextual bias” [47] in the economics domain. Hotly debated in the literature of social sciences [45, 48, 49], the crucial question is whether generalizations should be merely limited to qualitative behavioral insights when certain data patterns are observed, or the analyst can also go beyond that and even make reliable quantitative extrapolations. The question of generalizability has been greatly discussed in economic literature (as well as in other areas of social sciences) and has been described as the most fundamental question in the experimental economics [48]. This discussion will become even more fruitful in this particular topic by taking into consideration the certain context-specific elements that do exist in the real-world evacuations but are failed to be replicated by experimenters in vast majority of the virtual experiment settings. Because of the lack of objective comparative investigations connecting these two types of observations, as well as the sensitivity of the topic and its potential implications, researchers in this area have remained overly skeptical and cautious about authenticity and external validity of the findings obtained from hypothetical-choice observations collected in virtual evacuation studies [50, 51]. On the other hand, (framed or controlled) field experiments are regarded as a more plausible form of collecting empirical observations. They have been thought of as a great compromise between “controlled variation” and “realism” in this context, similar to their description in the economics literature as “an attractive marriage” between lab and real world [49].

The question of external validity, to our knowledge, has particularly remained unaddressed in the context of evacuation modeling due to other paucity of the real-choice data. Here, we make a pioneer attempt in addressing the aforesaid problem by linking between two types of empirical choice observations. We report on a simple laboratory-type experiment of hypothetical emergency exit choices and their imitations in a more realistic setting in action (i.e. a framed field-type experiment). Two exit-choice datasets are generated accordingly, “stated” or “hypothetical choices” (HC) and “revealed” or “real choices” (RC) of emergency exit.

The hypothetical exit scenarios were simply presented to participants as pictures. Participants were sampled from the pedestrians who exited a particular building, to which the hypothetical scenarios were referred (i.e. decisions were hypothesized to be made in an imaginary emergency exit occurring at the same building which the participant had just left (in a normal situation). We interviewed 169 individuals and each participant responded to 14 hypothetical exit scenarios. This provided us with a dataset of 2338 HC observations.

In the field, similar scenarios were then replicated in a more realistic fashion by conducting a number of mock (i.e. simulated) evacuation trials in an artificially-built model of (the floor level of) the same building depicted in the abovementioned pictures. Paid participants were recruited to perform the instructed evacuation tasks in the model building (up to 150 individuals performed each trial scenario). The experiments were recorded and then the escape movement of each participant was analyzed individually. Their movement trajectory was extracted using specialty software. The trajectory, body movement and head orientation of each participant was closely inspected to identify the likeliest moment their decisions were made. This provides us with a data set of 3015 RC observations. Econometric models were then estimated on each dataset.

For the modelling, we employed a well-established class of econometric models known as discrete-choice models to infer from individuals’ choices their evaluation, perception and prioritization of different attributes that contribute to their navigational decisions. We estimate fixed-parameter multinomial logit (FP-MNL) and random-parameter multinomial logit (RP-MNL) models as two well-practiced axiomatic paradigms of modeling human choices conceptualized by the notion of random utilities. A joint model estimation is also performed on the combined data using a generalized multinomial logit (GMNL) method [52] in order to identify possible parameter scale differences between the estimates inferred from the HC and RC contexts (as a measure of the randomness of decisions made in each context).

## Methods

### Hypothetical-choice experiments

The HC experiments were carried out during March and April 2014. The experimental procedure was approved by the Monash University Human Research Ethics Committee to which the authors were affiliated at the time of conducting the experiments. All methods were carried out in accordance to the approved guidelines. Also informed verbal consents were obtained from all subjects who accepted to participate in the experiment. The verbal consent method was chosen in order to facilitate the process of interview considering that the interviews took place at public places, and this was approved by the abovementioned ethics committee so long as the interviews were kept anonymous which was the case in this experiment. Participants were compensated for their time at the end of the interview by a modest amount of cash.

**Fig 1:**
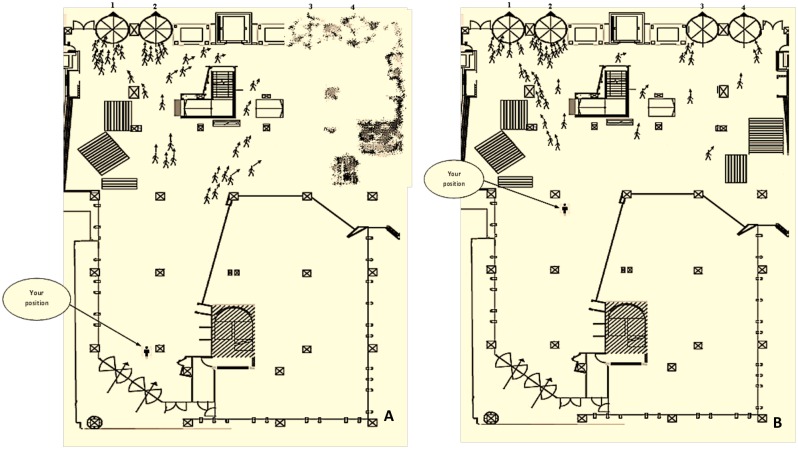
Samples of hypothetical exit-choice scenarios. Each scenario offers four alternative exits whose attributes compete with one another other. The decision maker is asked to make a trade-off between those factors in each scenario and choose the exit that they would choose in an evacuation scenario. (A) Sample of a hypothetical scenario in which two exits are invisible to the decision maker from their current position due to the presences of obstacles (a choice scenario with partially-ambiguous attributes for a subset of the choice set). The invisible exits are presented in a blurred way indicating that the level of congestion around those alternatives is not given to the decision maker. (B) Sample of a scenario in which all four exits are visible from the current position of the decision maker (a fully unambiguous choice scenario).

We interviewed pedestrians as they exited a building in Melbourne, Australia. Participants were sampled from exiting pedestrians. They were invited to a survey in which they were introduced to 14 hypothetical exit-choice scenarios. The choice scenarios presented a tradeoff between certain factors that are assumed to affect the choice of exit during an emergency evacuation (the design variables). The choice scenarios assumed that the evacuation is taking place in the floor level of the same building that the participant had just exited. The hypothetical position of the subject (as well as the other attributes) was shown in each scenario and the subjects were asked to hypothesize the presented decision situation as an emergency scenario and choose the exit that they would choose if an emergency egress arose in that building. Fig 1 illustrates two examples of the hypothetical choice scenarios. It was assumed that the escape was only possible through one of the four exits labeled as 1–4 in the figure. Thus the decision situations were framed as a choice between four exit alternatives.

The variables (exit attributes) whose relative weights are quantified by our models are: the number of evacuees “congesting” around each exit area (CONG), the spatial “distance” of the decision maker to each exit (DIST) (measured in meters), whether the exit is “visible” or “invisible” to the decision maker (VIS) (a 0–1 categorical variable with 1 corresponding to the visible exits), and the number of moving pedestrians (the size of directional flows) headed to each exit (denoted as FLTOEX, standing for “flow towards exit”). We distinguish, in our modeling, between directional flows based on the visibility of the exit to which those flows are headed. We label the flows headed towards visible and invisible exits as FLTOVIS and FLTOINVIS, respectively. When the exit is invisible as a result of the presence of architectural obstacles, the congestion level associated with that exit is not presented to the decision maker (Fig 1(A)) but those exits were allowed to still be chosen by the participants.

In total, 28 scenarios were designed and were divided into two blocks of 14 scenarios each. The participants were randomly assigned to one of the two blocks. Thus each subject only responded to 14 choice situations. The reason for generating multiple blocks (as opposed to one single block of 14 scenarios) was to create broader attribute range and greater attribute variability in the HC data that is essential for efficient parameter estimates (see [53] for a more detailed discussions on the variability issue). All 28 choice scenarios can be found in S1 Fig. A minimum sample size calculation was also carried out before sampling according to [54] to ensure a sufficient number of observations is gathered. In total, 169 individuals were surveyed resulting in a data set of 2338 HC observations. The spreadsheet file containing this dataset can be accessed in S1 Table.

There were also a few reasons we chose interviewing participants in the location to which the scenarios referred (i.e. the building whose map appeared in Fig 1) over the conventional computer-based counterpart experimental method conducted in the form of online surveys. An important difference between experiments of this type and many experimental games in economic studies is that there is no financial consequence associated with the decisions that individuals make in these choice experiments. In best cases, fixed modest monetary incentives are offered to each participant regardless of their responses to the experimental scenarios (as was the case in our experiments). Hence, there is always a concern that unreliable responses may be provided as a result of participants’ inattention or lack of interest or motivation [55, 56]. It is believed that by linking the hypothetical scenarios to a similar decision context that participants had just experienced in real life and of which had a clear memory and also by articulating and explaining the purpose and importance of the experiments on a face-to-face basis, participants are likelier to relate more realistically to the experiments [57]. Recent studies have provided emerging evidence suggesting that idea of referencing HC choice experiments relative to a real experience has offered promise in the derivation of reliable estimates and mitigation of the hypothetical bias [47]. Also, we assumed that participants’ inattention was likelier to be prevented as a result of being scrutinized by the interviewer who tried to keep them engaged [56]. Also, the egress geometry was likelier to be perceived more realistically and accurately as a result of participants having a clearer memory and perception of the location to which the scenarios referred. In another publication we quantified the extent to which this method succeeded to reduce the variance of random noises in the resultant models compared to a sample collected on a purely-random basis from students in lecture classes (where we eliminated the element of face-to-face interviews and referencing to the real-choice context) [58].

### Field-type (realistic-choice) experiments

Simulated emergency egress scenarios similar to what hypothesized in the HC experiments were replicated more realistically in a simplified and scaled model of the same building as depicted in the HC scenarios. The detailed map of the artificial model building can be found in S2 Fig. The experiments were conducted on 23^rd^ of February 2015. The experimental procedure was approved by the Monash University Human Research Ethics Committee to which the authors were affiliated at the time of conducting the experiments. All methods were carried out in accordance to the approved guidelines. Also informed consents were obtained from all subjects who accepted to participate in the experiment. The participants were invited using electronic mail invitation letters and used an online registration system to accept the invitation whereby they provided written consent as part of their registration. The registration procedure was also approved by the abovementioned committee. Participants were catered for and were also compensated for their time at the end of the trials (that lasted from about 10:00 a.m. to around 3:00 p.m.)

One hundred and fifty participants were hired to conduct the trial tasks. In total, 20 trial runs were carried out treated as emergency scenarios, with each scenario being a combination of certain design factors including the number of evacuees (either 75 or 150), the quantity of the available exits (2, 3 or 4), the position (i.e. spatial distribution) of the exits and the width (i.e. capacity) of the exits (either 50 cm or 100 cm). Full details of the trial runs can be found in S2 Table.

**Fig 2:**
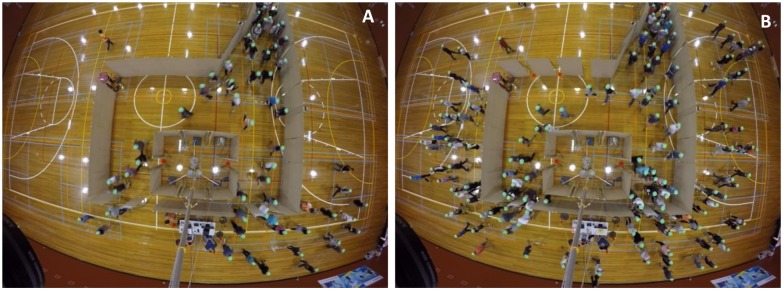
Snapshots from the raw footage of the mock evacuation experiments. A camera at 8m height above the floor records the experiments. The obstacle inside the room creates exit invisibility similar to the conditions in the actual building represented by the artificial model. Participants start evacuating at the entrance and are asked to exit the model building as quick as possible through one of the exits available to them. (A) A lightly congested treatment (75 evacuees). (B) A congested treatment (150 evacuees).

Participants were instructed to wait at the initial position of the evacuation room (See S2 Fig) and upon the start of each run, they were asked to enter the model building compete with others to escape from the room as quickly as possible assuming that they were running away from an acute threat (no specific type of threat was mentioned). The initial positions were randomized from run to run so that different individuals face different choice situations at each run (i.e. to avoid certain individuals locating in front of the crowd every time and thus not facing much congestion in any of their choices). They were kept motivated by the experimenters to keep running and looking for best escape options using verbal messages that repeated throughout each run. A square-shape obstacle positioned inside the room replicated a major architectural barrier that was present at the corresponding position in the real building. The participants were not aware of the exact configuration and the setup of the model building at each run in order to avoid a habituation effect, however they knew that there will be multiple escape alternatives (i.e. exits) at each run and that they will be able to find exits at both sides of the obstacle inside the room. A camera located at 8 meters height above the floor (whose legs were positioned inside the aforementioned square obstacle) recorded the trials and subjects’ movements. Fig 2 shows the snapshot samples from the raw footage of the two trial runs. Also, the raw footage of the two of the trial runs have been sampled and can be found in S1 and S2 Videos.

We had the participants wear colored beanies to facilitate the automatic recognition and tracking of their positions inside the evacuation room with the software that we used for this purpose. We used a specialty software called PeTrack [59] developed by the fourth author of this paper particularly for the analysis of the pedestrian movements and extraction of their trajectories under experimental conditions. The parameters of the software were calibrated according to the conditions of the experiments in the field (further details are provided in S1 Text).

The trajectory of the movement for each individual appeared in these scenarios was extracted. One major challenge that we faced in terms of converting the experimental observations to choice data was identifying the moments at which individuals had made their decisions. This clearly did not pertain to the HC experiments as the subjects had to make decisions at situations pre-specified by the experiment design in that case. Here, however we had to resort to any available external clue and indicator in order to identify the decisions. In doing so, in addition to the trajectory of their motion, their body movement and head orientation were closely examined as they moved to identify the likeliest moments when each subject made their exit decision. For this purpose, we attended to the movement of each subject individually looking for the moments after which they did not considerably explore different alternatives. This was typically accompanied by consistency in their body movement and their trajectory (e.g. no major change of movement direction). We defined such moments in the subjects’ movement as their “decision moment”. Since the process of choice extraction required human judgment (as described above), we decided not to automate the choice extraction phase, thus all the observations were collected manually by inspecting every single subject’s movement in the PeTrack environment.

The data extraction process was executed by 11 coders in total, including the first and the third author of this article. In total, 9 paid coders (6 undergraduate and 3 postgraduate students from the institute of the first three authors) were recruited and trained to perform the data extraction procedure (that in total demanded nearly 500 hours of work). Multiple training sessions in groups of 4–5 were practiced in order to ensure consistency between the works of different coders. Yet, as the decision identification process could be subject to the judgment of the coders, the sensitivity of the inferred parameter estimates was examined during the preliminary data analyses by performing model estimations on different blocks of the observations generated by different coders. An acceptable level of inter-sample variations was observed between the data segments provided by different coders indicating of no systematic bias engendered by the judgment of individual coders.

Also, another matter to be taken into consideration in relation with this issue is that minor errors in finding the decision moments are not supposed to make a drastic difference considering that the dynamics of the evacuation space (and as a result the attributes of different alternatives, relative to one another) do not show rapid variations over incremental amounts of time. In more specific terms for example, the exit that is most congested at time t is very likely to remain the most congested exit at times t+Δt or t-Δt as long as Δt is a relatively small increment. The same concept applies to the relative distances of the subject to different exits, or the visibility status of the exits to the subject. However, it might be the case that the flows moving to an exit at time t may turn into congestion around that exit at t+Δt and vice versa. We regard this as an aspect of the data extraction procedure that may be most sensitive to the identification of the exact decision moments.

**Fig 3:**
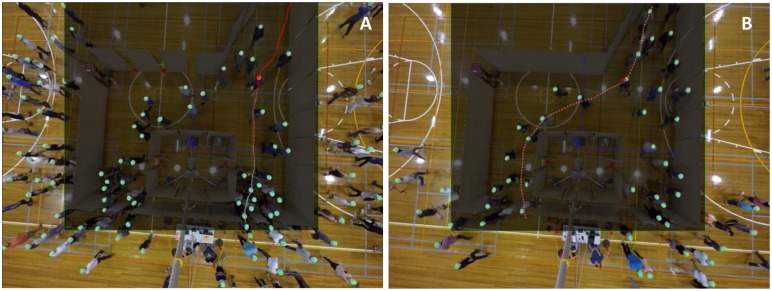
Raw footage of the trials is analyzed using specialty software calibrated for the specific environmental conditions in the field. Individuals are recognized and tracked by the software based on the color of the beanies they wore during the experiments. Trajectory, body movement and head orientation of each participant are inspected as external indicators to identify their likeliest decision moments. Decision moments are extracted as the moments after which participants showed most certainty and consistency in their movements. (A) A subject who chose exit 2, at their identified decision moment. (B) A subject who chose exit 3, at their identified decision moment.

Fig 3 exemplifies two subjects and their trajectories at their identified decision moments. For some subjects who clearly made more than one decision (individuals who made an initial decision, progressed towards it and then changed their choice (often in response to the excessive congestion developed around their chosen target)) more than one choice observation was extracted.

**Fig 4:**
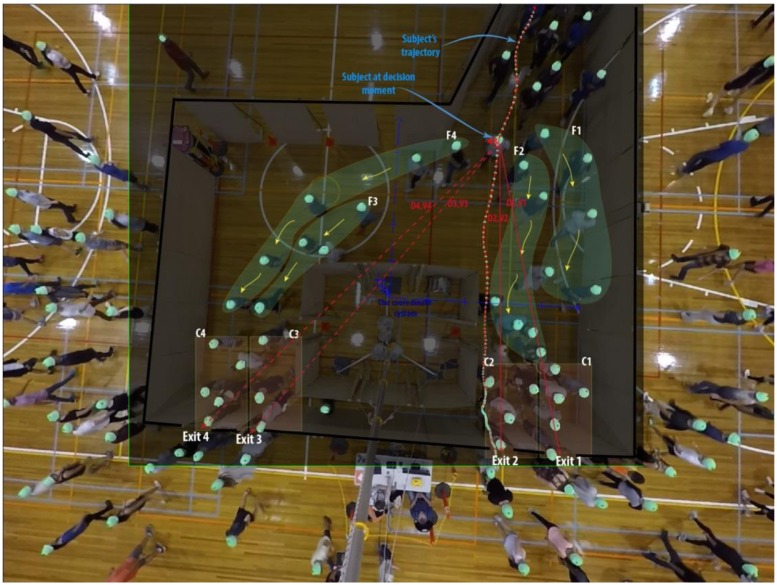
Extracting RC observations from the field-type evacuation experiments. A subject is shown at their decision moment along with their full trajectory of movement. The choice set and attribute levels of all alternative exits are recorded at the moment of the decision which together with the chosen alternative (here, Exit 2) constitute a single choice observation. The attributes that are recorded include C1–C4 that signify congestion (CONG) around each exit (the number of evacuees congregating near each exit), F1–F4 that denote the size of directional flows (the number of evacuees) moving to each exit (FLTOEX), V1–V4 as binary 0–1 variables representing visibility status of each exit (which equals 1 if the exit is visible from the position that the decision is made), D1–D4 that represent the subject’s distance to each exit, calculated based on the coordinates of the subject at their decision moment and measured in meters.

Once the decision moment was identified, the chosen alternative (i.e. the dependent variable), the choice set and the attribute levels of all alternatives (i.e. the independent variables) were recorded as a single “choice observation”. The measured variables were exactly the same variables as discussed previously for generating the HC scenarios. Fig 4 illustrates more details on how attribute levels were measured at extracted decision moments. A data set of 3015 RC observations was collected in total. The serial correlation effect (the fact that the same group of people made decisions and contributed observations to the data from run to run) had to be downplayed in the RC data considering that recognition of individuals’ identity between different runs was impossible from the footage (as participants all wore similar beanies). They could only be recognised individually within each run, and this effect has been embodied by the RC data (clearly, for any individual who contributed multiple observations in each single run). The extracted data file can be accessed in S3 Table.

### Analysis and Modeling Method

Individual n’s choice among J_n_ alternatives at choice situation t (t = 1, …, T_n_), is the one with maximum utility, where the utility functions are as Eq 1, consisting of a deterministic part V_nit_ and a random disturbance part ε_int_. The vector **x**_nit_ is the vector of all attributes that appear in all utility functions (Eq 2) (defined previosuly), ε_nit_ represents random unobserved factors of utilities, **β**_n_ is the vector of utility coefficients (with probability density function denoted as g(**β**_n_)), and θ_n_ is the utility scale factor. The coefficient vector is specified as **β**_n_ = **β** + **Γω**_n_ where **β** is the vector of means of coefficients, **ω**_n_ is a vector of independent standard normal variables, and **Γ** is the Cholesky factor of the covariance matrix, **Σ = ΓΓ′** (see [60] for an exact definition). Random unobserved factors are assumed to be distributed identically and independently as (standard/normalized) Extreme Value Type 1 with the density function given as $\text{f}\left(\varepsilon_{\text{int}}\right)={\text{e}}^{-\varepsilon_{\text{int}}}{\text{e}}^{-{\text{e}}^{-\varepsilon_{\text{int}}}}$ (see [61, 62] for further information about the characteristics of this distribution). The scale factor is also specified as $\theta_{\text{n}}={\text{e}}^{{\delta \text{z}}_{\text{n}}}$ with z_n_, the scale dummy variable, being a 0–1 binary variable that equals 1 when the choice by individual n has been made in the HC context. The above specification relates to the GMNL model where the two datasets are pooled (i.e. HC+RC data). The model reduces to RP-MNL when δ = 0, and will collapse further to FP-MNL when δ = 0 and **ω**_n_ = **0** at the same time.

The probability of the individual making the same sequence of choices they observed to have made in the data ***i*** = 〈*i*_1_, …, *i*_*T*_〉 is given in Eq 3 and is introduced to the likelihood function as the computational term associated with the information obtained from that individual. The log-likelihood function (LL) is defined in Eq 4 and the estimates are inferred from a simulated maximum log-likelihood procedure. The goodness-of-fit measure also known as pseudo rho-squared is defined as the ratio of the improvement in log-likelihood after reaching the convergence (i.e. at maximized log-likelihood, LL*) relative to its initial value (i.e. where all parameters are set at zero, LL^**0**^) (Eq 5).

$${\text{U}}_{\text{nit}}={\text{ V}}_{\text{nit}}+\varepsilon_{\text{int}}=\theta_{\text{n}}\beta_{\text{n}}^{\text{'}}x_{\text{nit}}\;+{\;\varepsilon }_{\text{int}}\text{ i}=1,\ldots ,{\text{J}}_{\text{n}}$$ (1)

$$x_{\text{nit}}=\left({\left(\text{DIST}\right)}_{\text{nit}},\;{\left(\text{CONG}\right)}_{\text{nit}},\;\underset{={\left(\text{FLTOVIS}\right)}_{\text{nit}}}{\underset{︸}{{\left(\text{VIS}\right)}_{\text{nit}}*{\left(\text{FLTOEX}\right)}_{\text{nit}}}},\underset{={\left(\text{FLTOINVIS}\right)}_{\text{nit}}}{\underset{︸}{{\left(1-\text{VIS}\right)}_{\text{nit}}*{\left(\text{FLTOEX}\right)}_{\text{nit}}}},{\left(\text{VIS}\right)}_{\text{nit}}\right)$$ (2)

$$\begin{array}{l} {\text{P}}_{\text{nit}}=\int_{\beta_{\text{n}}^{\;}}\overset{\text{T}}{\underset{\text{t}=1}{\prod }}\frac{{\text{e}}^{\theta_{\text{n}}\beta_{\text{n}}^{\text{'}}x_{{\text{ni}}_{t}\text{t}}}}{\sum_{\text{j}=1}^{{\text{J}}_{\text{n}}}{\text{e}}^{\theta_{\text{n}}\beta_{\text{n}}^{\text{'}}x_{\text{njt}}}}\text{ g}\left(\beta_{\text{n}}^{\;}\right)\text{d}\beta_{\text{n}}^{\;} \end{array}$$ (3)

$$\text{LL}=\sum_{n}\text{ln}({\text{P}}_{\text{nit}})$$ (4)

$$\rho^{2}=1-\frac{{\text{LL}}^{*}}{{\text{LL}}^{0}}\;$$ (5)

## Results

**Fig 5:**
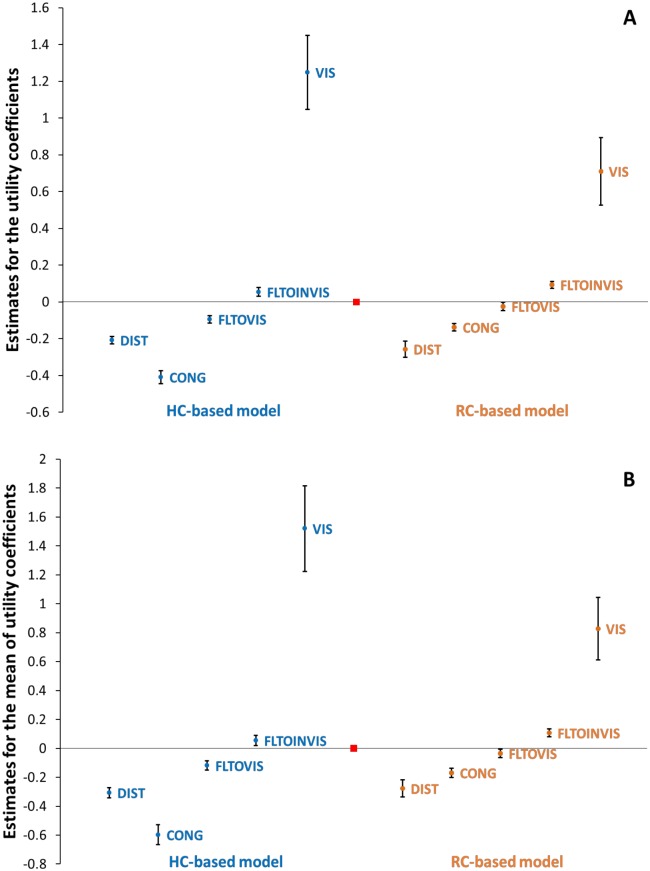
Comparing the model estimates obtained from the HC against RC data. The error bars represent the 95% confidence interval of each estimate. The red square separates the HC-based estimates from those obtained from the RC observations. (A) Estimates obtained from a FP-MNL model specification. (B) Estimates obtained from a RP-MNL model specification (the mean of utility coefficients). Note that according to the scale difference of the models derived from the HC and RC segments, comparison between the corresponding “magnitude” of the individual estimates between the HC and RC contexts would not be meaningful and is not intended by these graphs. Only, the sign, significance and the estimate patterns of the parameters are meaningfully comparable here.

Table 1 provides the result of the parameter estimates using the two models (i.e. FP-MNL and RP-MNL models) specified in the previous section as well as the joint estimation results (i.e. the GMNL model). Also, Fig 5 provides a visual presentation of the mean of the estimates by depicting their 95% confidence intervals. In terms of the goodness of fits, the models estimated on RC observations prove to have provided a substantially better statistical fit to the data. Here in the followings, we put into contrast the models estimated on the two different observation types from a number of relevant econometric and behavioral perspectives.

**Table 1:** Model estimation results on HC and RC datasets, and the combined (RC+HC) data.

Parameter	Estimate				
	FP-MNL model		RP-MNL model		GMNL model
	HC data	RC data	HC data	RC data	RC+HC data
**Means of utility coefficients**					
DIST	-0.208 (-21.18)***	-0.256 (-11.40)***	-0.306 (-16.88)***	-0.277 (-9.08)***	-0.096 (-17.94)***
CONG	-0.409 (-22.66)***	-0.138 (-12.90)***	-0.597 (-17.13)***	-0.169 (-10.02)***	-0.178 (-20.07)***
FLTOVIS	-0.094 (-9.06)***	-0.024 (-2.07)**	-0.119 (-7.22)***	-0.035 (-2.37)**	-0.048 (-10.84)***
FLTOINVIS	0.054 (4.53)***	0.093 (9.52)***	0.055 (3.04)***	0.107 (7.75)***	0.047 (6.98)***
VIS	1.249 (12.14)***	0.710 (2.85)***	1.520 (10.06)***	0.828 (7.49)***	0.791 (15.26)***
**Standard deviations of coefficients**					
DIST			0.171 (9.90)***	0.091 (0.93)	0.574 (4.02)***
CONG			0.327 (11.71)***	0.181 (4.48)***	0.325 (2.58)***
FLTOVIS			0.134 (9.27)***	0.070 (2.92)***	0.112 (0.79)
FLTOINVIS			0.136 (9.30)***	0.072 (2.99)***	0.115 (0.80)
VIS			1.190 (6.04)***	0.091 (0.42)	0.068 (0.07)
**Utility-scale dummy variable**					0.696 (12.48)***
**Statistical fit measures**					
Number of choice observations	2338	3015	2338	3015	5353
Initial log-likelihood	-3241.16	-4174.13	-3241.16	-4174.13	-7415.29
Optimum log-likelihood	-2855.16	-2863.54	-2610.42	-2854.74	-5798.84
Pseudo ρ^2^	0.119	0.314	0.195	0.316	0.218

Note that *** and ** indicate statistical significance at 99% and 95% level respectively. The values in parentheses show the t-statistic of the estimates.

### Sign and significance of the estimates

Models estimated on both HC and RC datasets show a general agreement between the two observation types as similar estimation patterns are clearly observable (Fig 5). There is a perfect agreement between the HC and RC-based models in terms of the sign of the estimated coefficients as well as their statistical significance (of the means). All the variables specified in our models proved to be statistically meaningful at highest conventional statistical levels. As intuitively expected, the distance (DIST) and congestion (CONG) factors both impact negatively on the utility of the exits, and visible exits generate higher utilities than invisible exits (reflected in the positive mean estimate of the VIS variable).

Both types of observations also agree upon an important estimation finding suggesting that the perception of observing the directional (or moving) crowd flows by decision makers significantly depends on the visibility of the exit to which that flow is moving. The hypothesis that the utility coefficients associated to FLTOVIS and FLTOINVIS variables are equal is rejected (by both of our datasets and both FP-MNL and RP-MNL modelling methods) at conventional confidence levels. In other words, it is strongly suggested by all the estimated models that flows of evacuees are perceived as negative utility elements when the exit to which that flow is headed is visible to the decision maker. Whereas, such flows are suggested to (on average) add to the perceived utility of the exit when the decision maker themselves cannot see the exit targeted by the flow.

### Following versus avoiding others

Strongly suggested by both observation types, the recent finding discussed above (i.e. the difference between how moving flows are perceived based on the visibility of their targets) has important implications as to our understanding of the role played by the social influences in making emergency navigational decisions which is often referred to as “herding” or “follow-the-crowd” behavior in the literature [3, 4]. There has been a widespread conventional belief in the literature (often based on pure speculations and anecdotal evidence) as to the possibility of the formation of a dominant tendency during evacuations to follow what others do for their escape [12].

Important relevant fundamental questions, however, are yet to be answered based on concrete empirical evidence. It is still not known with certainty whether the herding behavior per se can be considered as a sole determinant of the evacuees’ escape decisions, nor are the role of context-specific factors—that may strengthen or moderate this tendency—adequately understood. Also, it is not clear whether the tendency to follow others is a global behavior among all individuals involved in an emergency or it only is exhibited by a portion of evacuees (i.e. the problem of preference/perception heterogeneity). Is it, for example, reasonable to classify evacuees as those who do and those who do not follow others, and even if so, how the population is split on that account.

The relation between the social influence and decision making in general has been vastly studied and discussed in the social sciences. The existing literature indicates a substantial level of context-specificity attached to the problem. For example, Faria et al. [63] found out based on a series of field-type experiments that, similar to groups of animals [64], when reaching a target, individuals of a group are capable of identifying informed individual(s) without the aid of obvious signaling and follow them to enhance the group performance. Gallup et al. [65] showed how visual attention of pedestrians in a crowd can in limited ways be diverted by few individuals gazing up at a certain point. Also, in a series of computer-aided human experiments Zhao et al. [66] showed that when humans compete for limited resources in a complex adaptive system, the effect emerged from the formation of herding can vary from beneficial to detrimental depending on the biasedness of the resource allocations.

Despite the aforementioned evidence, there have been limited and scattered investigations of this phenomenon in relation with the navigational decision-making of human crowds during evacuations. Useful empirical evidence, however, is provided by the work of Bode and Codling [38] and similarly by Bode et al. [31] who suggested that subjects familiarized with the egress environment do not develop a strong tendency to follow others when they can see the entire egress environment, even under stress-induced treatments in the lab. In that spirit, our model estimates are to a great extent consistent with their findings. However, what is further suggested by our experiments is that, firstly, exit choices are not likely to be solely determined by a single factor (such as herding) and rather, there proves to be a combination of factors among which tradeoffs are made (of which following/avoiding others is only one single element). Secondly, it is manifested in a quantitative fashion by our model estimates that a significant dependency exists between individuals’ tendency to follow other evacuees’ decisions and the decision maker’s knowledge (or awareness) of exit attributes (such as the level of congestion around the exits). Results from both experiment types clearly suggest that attribute unawareness increases the likelihood of herding behavior to be displayed. In other words, attribute unawareness (introduced to our trials in the form of exit invisibility) can entirely change the direction at which observing the decisions of moving flows of evacuees impact on individuals’ decisions from a negative utility element. As a result of the attribute unawareness (or attribute ambiguity), the impact of the majority’s choice can alter from being perceived as potential congestion (thus, increasing the likelihood of avoiding others) to extra sources of information (thus, increasing the likelihood of conforming to the crowd).

### Perception of congestion relative to distance

Referring to Fig 5, one major difference between the HC and RC estimates is the relatively-smaller (absolute) magnitude of the estimate for the coefficient of congestion (CONG) in the RC models compared to those of the HC models. The difference is even clearer when comparing the positions at which the estimates of the coefficient of CONG variable stand in Fig 5 relative to the position of the coefficient for distance (DIST) variable. At a possible explanation, we attribute this to a possible systematic bias in terms of the perception of distance (relative to the perception of congestion) in the HC experiments. When making the exit decisions in the HC experiments, subjects were likelier to underestimate the impact of distances (relative to congestion) as they did not need to actually traverse those distances, whereas an actual effort would become necessary when the decision is made in the field. In other words, it appears that our HC experiment participants have placed less emphasis on minimizing the distance (relative to minimizing the congestion) and have instead found the congestion impact more salient on their decisions than the decisions of the participants in the field-type trials.

### Decision randomness and utility scales

Referring to the 95% confidence intervals presented in Fig 5, another clear difference between the estimates obtained from the HC observations compared to those derived from the RC observations is that the HC estimates appear to be generally more inflated in terms of their absolute magnitudes. This could be attributed to the difference in the scale of the utilities as a relevant factor that should be taken into consideration when comparing estimates of the two choice models derived from two different datasets. The scale at which the coefficients are estimated (relative to the scale of the random error components that is typically normalized to a fixed value [61, 62]) is a proxy and a measure of the randomness extent of the decisions. The smaller the estimates’ scale, the stronger the role played by the error components and as a result, the more random the choices [67]. From a statistical estimation point of view, the utility scale is not statistically identifiable (separately from the coefficients) based on a single dataset. However, utility scales associated with different data segments (when combining various sources of choice observations) can be estimated on a relative basis.

We quantified this effect by adopting the joint estimation method proposed by Fiebig et al. [52] known as the “generalized multinomial logit” (GMNL) that can distinguish between the estimated scale associated with different data segments when different data sources are pooled. We basically normalized the utility scale associated with the RC observations to unity (or equally, we normalized the utility scale dummy variable, δ, associated with the RC segment to 0) and estimated that of the HC segment relative to that.

Results (reflected in the statistical significance of the estimated coefficient for “utility-scale dummy variable” in Table 1) confirm that the RC observations are associated with smaller utility scales at a statistically significant level compared to the HC observations. This indicates that decisions in our field-type experiments have been made more randomly than those made in the hypothetical scenarios. In other words, the hypothetical setting seems to have underestimated how randomly people make their navigational escape choices. This clearly could be attributable to a stronger attribute perception error as well as less “time to think” [68, 69] when people make their choices in a real crowd compared to when they are provided with a top-down view of the environment and more time to decide in a hypothetical setting.

### Preference heterogeneity

The presence of the coefficient heterogeneity can be detected by the RP-MNL model estimates through inspection of the statistical significance of the estimated standard deviations. In this spirit, the model estimated based on the HC data suggests the presence of significant heterogeneity attached to all utility variables, whereas the model from the field excludes distance and visibility from this list and suggests decision makers as being statistically homogenous in terms of the relative weights they associate to these two utility factors. In this sense, one can say that the effect of heterogeneity has been to an extent overplayed by the HC observations.

### Model predictions

We quantitatively investigate the overall impact of all the differences that we highlighted in previous lines in terms of the estimates obtained from our real and hypothetical choice experiments on predictions of the models. Here, the question of interest is the extent to which predictions of the models derived from these two different forms of observations compare to one another. In other words, there proves to be certain similarities as well as dissimilarities between HC and RC-based models and it is still not clear to what degrees this makes predictions of the two models differ from one another.

**Fig 6:**
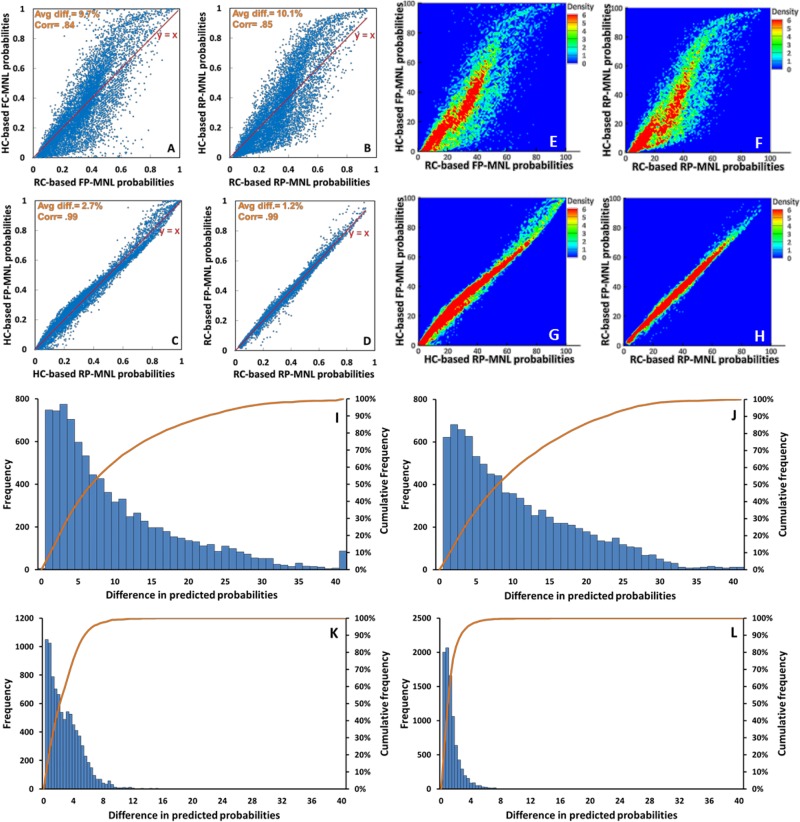
The impact of the context dependency (HC versus RC) and preference heterogeneity on the model predictions. Each point in the scatterplots (A)-(D) is associated with one alternative exit in one choice situation extracted from the field-type trials. The coordinates of each point are the probabilities predicted for that alternative (exit) by the model represented by the two axes. Figures (A) and (B) measure the effect of context dependency on predictions by making pairwise comparisons between the predictions of the counterpart models obtained from the real and hypothetical experiments. Figures (C) and (D) measure the effect of accommodating preference/perception heterogeneity on the predictions by making pairwise comparisons between the predictions obtained from models that can and cannot accommodate the heterogeneity effect (estimated on the same data). The average of absolute differences (Avg diff.) and the correlations (Corr) between each pair of data series are shown on each scatter plot. Figure (E)-(H) color-code the concentration of the points (the number of points per 1×1 square) in the scatterplots presented in Figures (A)-(D) respectively. Figures (I)-(L) show the distribution of the absolute differences between the predicted probabilities of each model pair compared in figures (A)-(D) respectively (i.e. the distribution of the absolute differences between the values of the x and y coordinates of the points shown in figures (A)-(D)).

To address this question, we excluded from the RC observations the element of the dependent variables (i.e. the choice) and set the resultant “choice situations” as the basis of comparisons (without making any use of the choices made by subjects in those observations). The probabilities predicted for each alternative exit in each observed choice situation by each of the four models presented in Table 1 are computed. The paired predictions are then compared and visualized through the scatter plots depicted in Fig 6(A) and 6(B). These figures visualize the points whose coordinates are the probabilities predicted by the two different models represented by their axes (for example, an RC-based model and its counterpart HC-based model) with the y = x line superimposed on the scatterplots to facilitate the comparison. The scatter of the points around the bisector line is a visual measure of how closely the two models represented by the two axes produce their probabilities. Also, the graphs shown in Fig 6(E) and 6(F) respectively correspond with the scatterplots shown in Fig 6(A) and 6(B) and color-code the concentration of the points in the scatterplot they represent. Fig 6(I) and 6(J) also visualize the distribution of the absolute differences between the predictions of the RC-based and HC-based models (i.e. the distribution of the absolute differences between the coordinates of the points visualized in the aforementioned scatterplots) using histograms.

According to these figures, there appears to be a substantial level of agreement between the predictions of the RC and HC-based models. We quantify this effect by computing the correlations and the average differences between each pair of data series presented in Fig 6(A) and 6(B). The correlation between the two data series presented by Fig 6(A) (i.e. the predictions of a FP-MNL estimated on the HC observations against those of its counterpart model estimated on the RC observations) is 84% and their (absolute) differences average 9.7%. According to Fig 6(I), 85% of these pairwise absolute differences in the predicted probabilities are smaller than 19% probability (the 85^th^ percentile). Similar statistics are obtained when the predictions of the random-parameter counterpart models are compared (average difference = 10.1%, correlation = .85 and the 85^th^ percentile = 20% probability). Also, the corresponding graphs shown in Fig 6(E) and 6(F) show that in each case there is much greater concentration of the points around the y = x line and the concentration drops substantially as we go further away from the bisector line.

We also isolate the effect of coefficient heterogeneity on the predictions by making similar comparisons between the predictions of the FP-MNL model (that does not accommodate the preference heterogeneity) against those of the RP-MNL model (that does accommodate the preference heterogeneity). The scatterplots shown in Fig 6(C) and 6(D), and the corresponding color-coded graphs in Fig 6(G) and 6(H) as well as the histogram of distributions depicted in Fig 6(K) and 6(L) visualize these pairwise comparisons. Results (again quantified in terms of the correlations and average differences shown on each graph) clearly suggest the negligibility of the difference that incorporation of the preference heterogeneity into our models has made in terms of their prediction performance (an average difference of 2.7% and a correlation of .99 between the predictions of the models derived from the RC observations, for example).

## Conclusions and Discussion

Discrete-choice models were estimated based on the exit-choice observations collected in two different experimental settings, choices made in response to hypothetical evacuation scenarios and choices made in mock evacuation trials that imitated the hypothetical setting in a more realistic fashion (using an independent sample of participants). The aim and motive of the study was twofold. The study primarily intended to investigate the external validity of the results obtained from the hypothetical decision-making experiments in the context of emergency wayfinding. In addition, prediction models of evacuees’ exit choices as well as important behavioral insights were gained from the experimental observations. Similarities and dissimilarities of the econometric estimates inferred from these two forms of choice observations were investigated in terms of the sign, statistical significance, magnitude, person-to-person variations, and behavioral interpretation of the estimated parameters, in addition to the utility scales (that reflect the context-dependence randomness of the decisions). Also, predictions of the models derived from the hypothetical and realistic experiments were contrasted.

The model estimation results manifested perfect consistency between the sign and statistical significance of the parameters obtained from the hypothetical and realistic experiments. Also fairly similar estimate patterns were observed when the estimates of the two models were visualized against each other. However, the analyses also highlighted certain discrepancies. The modeling results suggested that spatial distances have been underestimated in the hypothetical setting. Also, it became apparent that people have made their decisions in the realistic setting more randomly compared to the situation where they simply stated their responses to the hypothetical scenarios. Also, in the realistic setting, participants appeared to be less heterogeneous in terms of the relative weight they associate to the various factors contributing to their choice of exit, whereas their hypothetical responses had indicated a more pronounced person-to-person variation effect (although, further analyses later revealed that inclusion or exclusion of the coefficient heterogeneity effect plays a negligible role in our model predictions).

Nonetheless, comparative analyses highlighted that the overall effect of these discrepancies did not make a drastic difference in the predictions obtained from the models estimated on real and hypothetical choices. Models derived from these two observation types made fairly similar predictions and the type of data used for modelling did not on average make a dramatic difference in the prediction phase (the differences in the produced probabilities averaged nearly 10%). In other words, we could have made by-and-large same predictions using the hypothetical choice observations as we did based on the real choice observations.

The estimated models also shed interesting insights into the behavior of human crowds during escapes from confined multi-exit environments. Models suggested that the decisions of evacuees are made based on a tradeoff between a set of variables and single-variable-based decision criteria do not adequately explain their escape decisions. People make tradeoffs between minimizing spatial distances, choosing less congested areas and choosing visible exits, and are also influenced by observing the decisions of others. However, they evaluate the decisions of others differently depending on the presence or absence of ambiguity in the escape environment. We found out that moving flows of crowd headed towards a target that is visible by the decision maker are perceived negatively (i.e. potential congestions causing further delays) whereas similar flows are perceived as positive utility elements (in terms of the central tendency) when attributes of their target is ambiguous to the decision maker (as a result of the exit being invisible). In other words, the introduction of ambiguity proved to make a meaningful difference in the way that the social influence is perceived by decision makers. In more specific terms, flows of fleeing evacuees are not likely to trigger a herding effect when the decision maker is aware of the presence and the attributes of their targets, whereas attribute unawareness increases the likelihood of herding in making navigational decisions.

The models resulted from this study are fully generic in variables (i.e. we avoided any variable or major modeling element specific to our experimental setup). Accordingly, they can be readily introduced to a wide range of computer programs that simulate crowd evacuations. Among the models investigated in this work, application of the model estimated on the combined data might be preferred as it benefits from a larger sample of observations. In such case, application of the model at the scale associated with the real choice segment of the data would be recommended as it corresponds to a more realistic experimental setting. The resultant simulation program can be used to evaluate the performance and efficiency of evacuation scenarios in built environments.

However, there are certain limitations that need to be taken into consideration for potential prediction purposes. First of all, the validity of our models needs to be further scrutinized in larger venues than what we used for our experimentations. Moreover, the role of context-specific conditions under which our observations (and as a result, our models) were derived should also be taken into account for prediction purposes. That mainly includes occupants’ familiarity with the escape environment, the extent of the time pressure and urgency and the general visibility condition in the egress environment. It is implicitly assumed in our models that evacuees are at least partly familiar with the geometry of the escape environment. Acknowledging the well-established role of memory in navigational decision-making [70–72] we expect some behavioral differences when evacuation of human crowds who are unfamiliar with their surrounding environment is concerned. Also, although we took certain measures to make the field experiments as realistic as possible, the role of the time pressure and urgency level that might cause high levels of fear and stress in extreme emergency scenarios had to be downplayed in this work. We assume, it might be the case that as a result of an extreme level of urgency (e.g. acute fear state), people may attend to a smaller set of factors than what our models suggested and/or assign different relative priorities to those factors. Similarly, it could be hypothesized that when the general visibility condition is reduced in the escape environment, as a result of smoke for example, certain factors may play a more pronounced role in modulation of the navigational decisions and as a result, different behavior might emerge. In particular, considering our finding as to the strong association between the perception of the social influence and the ambiguity level in making navigational decisions, we assume that visually ambiguous evacuation situations could be a context where tendency to exhibit herding behavior can be stronger than what we observed in our experiments (in which the general vision was assumed intact). Investigating how changes in these context-specific factors may impact on the escape behavior can open many more research questions to be addressed by future studies. Yet at this stage, acknowledging potential consequences of misguided modeling in this safety-related topic, we should recommend cautions about extrapolating the models and findings of this study to the contexts to which they do not belong.

Also, the fact that we found similarities between the findings we earned from the hypothetical and realistic decision settings (more than the level we expected) does not necessarily indicate possible generalizations to all decision contexts, nor is it intended to downplay the importance of real-world data and field experimentation. Our findings could appear promising as to the potential relevance of the hypothetical choice observations when real or field data is prohibitively inaccessible (at least for the decision contexts of no financial consequence). Yet, the finding of this single study would hardly provide conclusive evidence for making robust generalizations in terms of the reliable applicability of this experimentation method to all contexts of decision-making. We see data collected in hypothetical and realistic settings as complements and our work was even intended to encourage collection of more field-type observations rather undermining their importance. We believe this may be the only way through which one can make solid conclusions as to the limits of the applicability and generalizability of the lab experiments. We believe that more comparative evidence of this type may help researchers to map out boundary conditions and the type of problems that fall beyond the range of the generalizability of the hypothetical decision experiments. In a recent meta-analysis study for example, Herbst and Mas [73] demonstrated great reliability of laboratory-generated findings in the context of “productivity spillover” at quantitative levels. The accumulation of such evidence might at least provide researchers with convincing grounds to rely on the idea of “some number better than no number” [49] in situations and contexts where no real observations are available to the analyst.

Also, as a different perspective it might be of the interest of cognitive neuroscientists to provide more evidence as to how differently real and hypothetical decisions are processed and encoded in the brain in order to explore the cognitive underlying differences of decision making in real versus hypothetical settings. To our knowledge, studies of this type have started to emerge [74] which could provide a new frontier and perspective for advancing the state of the knowledge on this problem.

## Supporting Information

S1 Fig Hypothetical exit-choice scenarios. (PDF)

S2 Fig The map based on which the artificial model building was made for the simulated evacuation trials. (PDF)

S1 Table Hypothetical exit-choice data, obtained from the survey experiment. (XLS)

S2 Table Details of the trial runs for the simulated evacuation experiments. (PDF)

S3 Table Realistic exit-choice data, obtained from the field-type experiment. (XLS)

S1 Text Details on the calibration of the software for trajectory extraction in the simulated evacuation experiments. (DOCX)

S1 Video Sample raw footage of the simulated evacuation trials, scenario #9. (WMV)

S2 Video Sample raw footage of the simulated evacuation trials, scenario #19. (WMV)
