# Supplementary material for: How Simple Hypothetical-Choice Experiments Can Be Utilized to Learn Humans’ Navigational Escape Decisions in Emergencies
Source: PLoS One. 2016 Nov 21;11(11):e0166908. doi: 10.1371/journal.pone.0166908 (PMC5117746; doi:10.1371/journal.pone.0166908)
Supplement: S1 Fig — (PDF) [file pone.0166908.s001.pdf]

Hypothetical choice experiment scenarios

SC (I) scenarios - Block (I)

Scenario 1

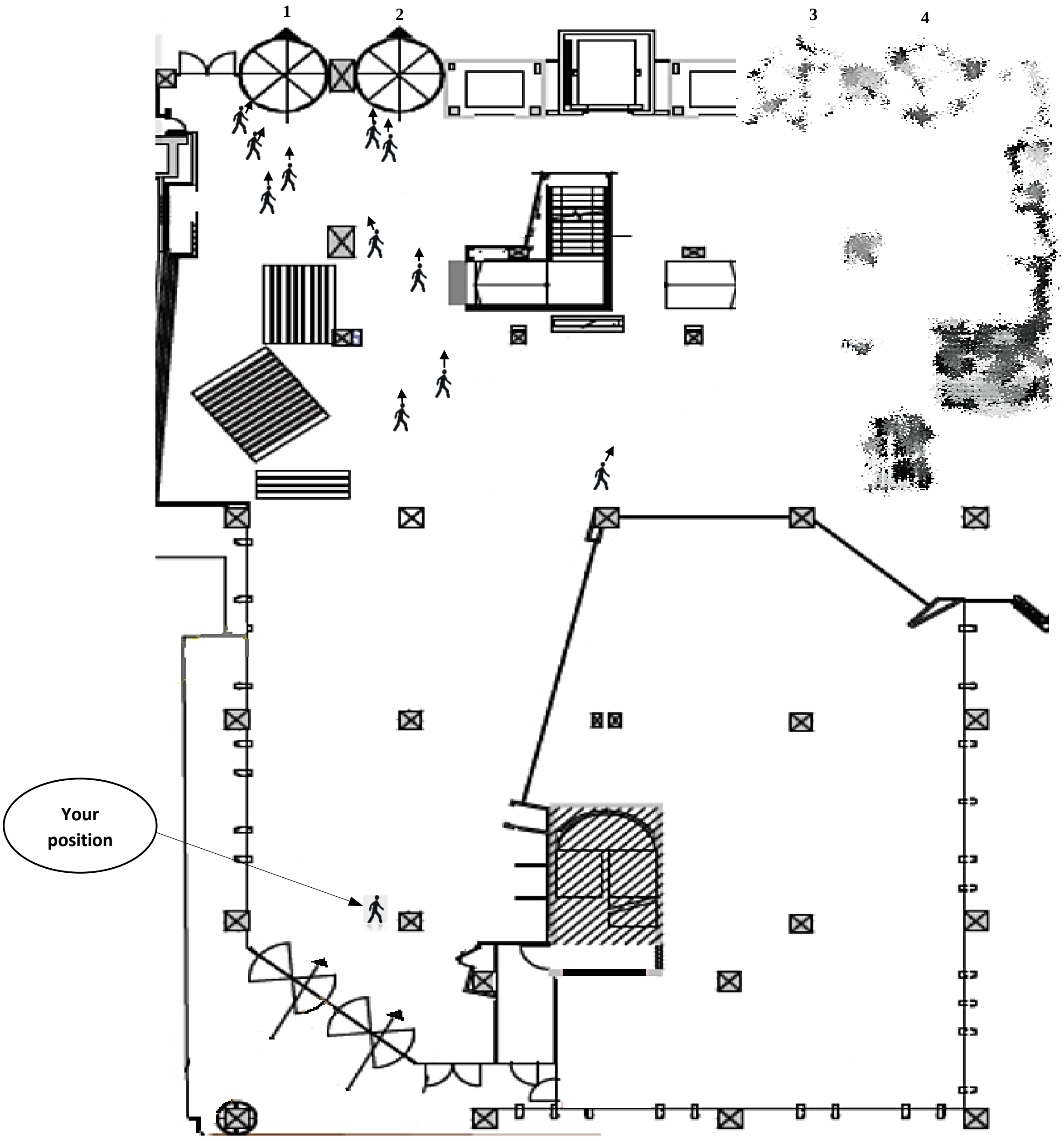

Your Choice:

Exit 1 Exit 2 Exit 3 Exit 4

Scenario 2

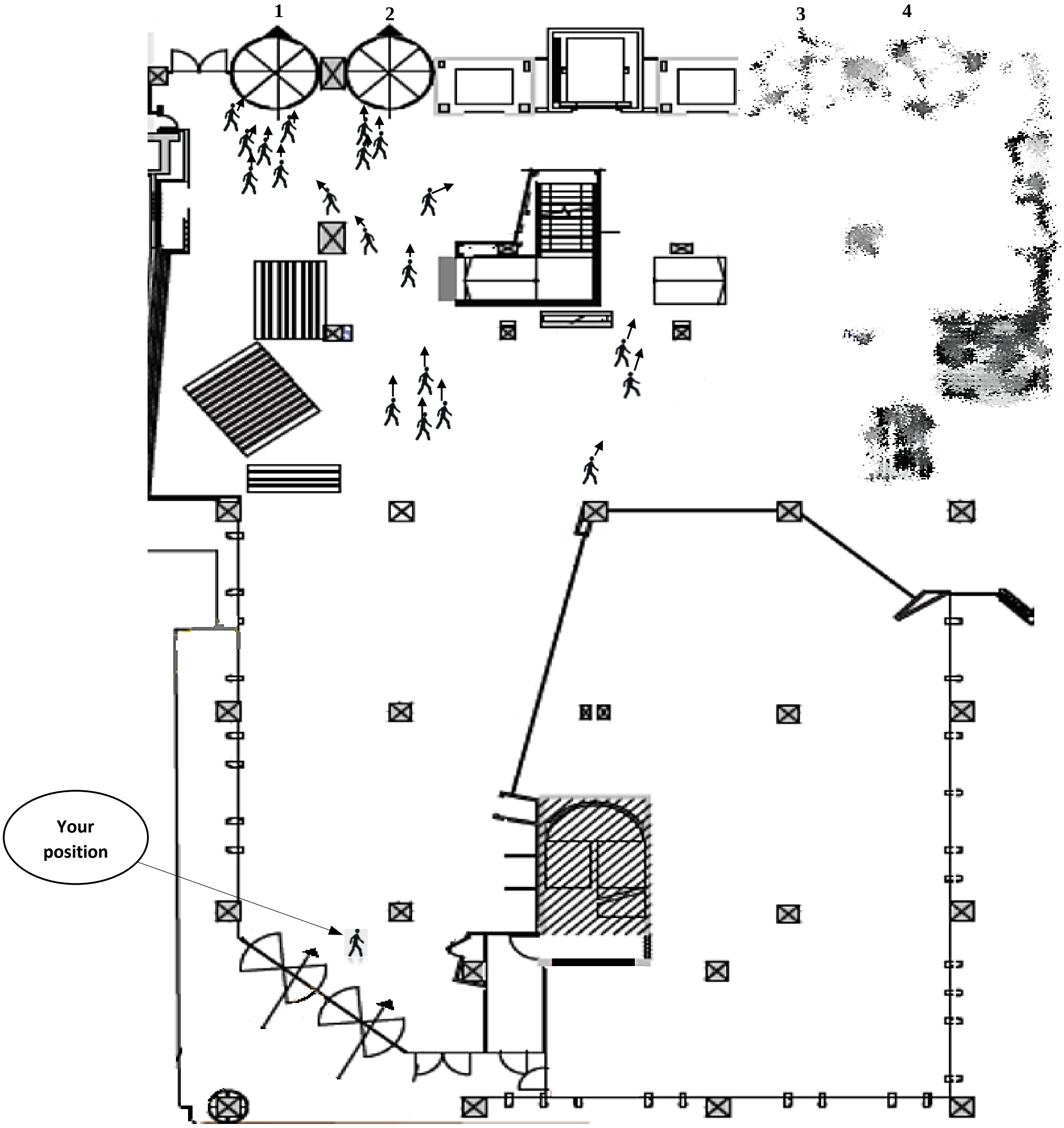

Your Choice:

Exit 1 Exit 2 Exit 3 Exit 4

Scenario 3

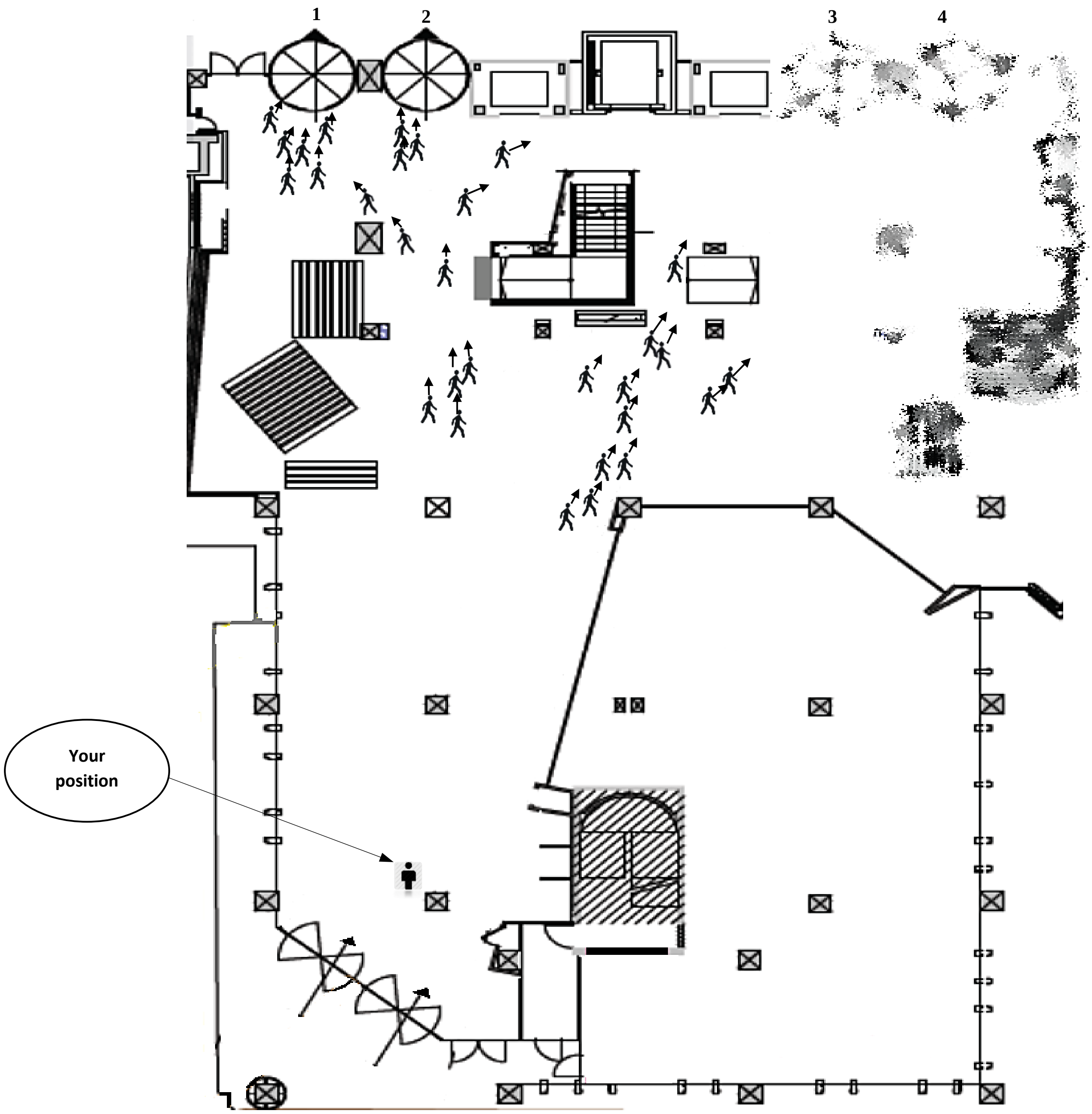

Your Choice:

Exit 1 Exit 2 Exit 3 Exit 4

Scenario 4

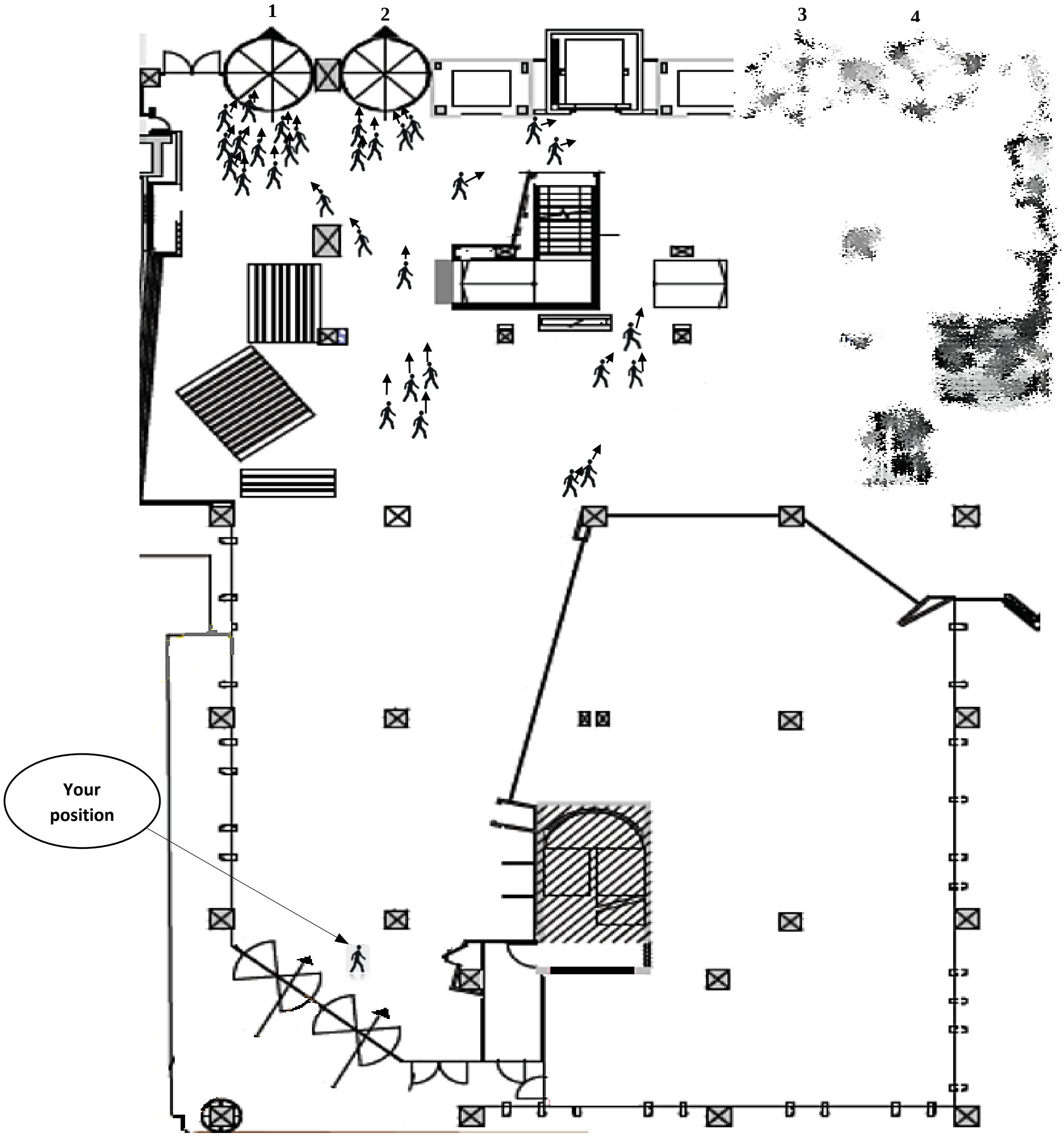

Your Choice:

Exit 1 | Exit 2 | Exit 3 | Exit 4

Scenario 5

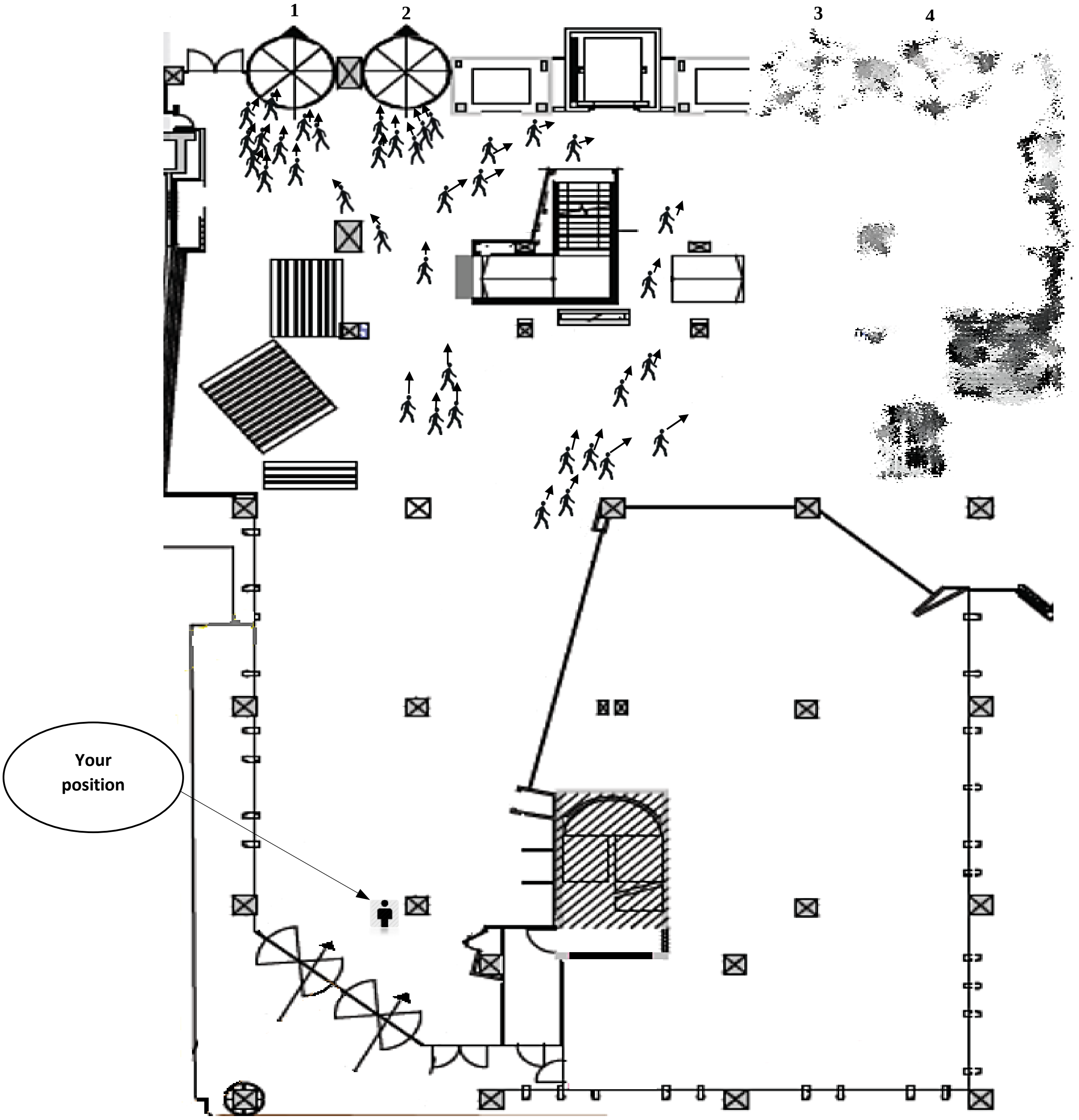

Your Choice:

Exit 1 Exit 2 Exit 3 Exit 4

Scenario 6

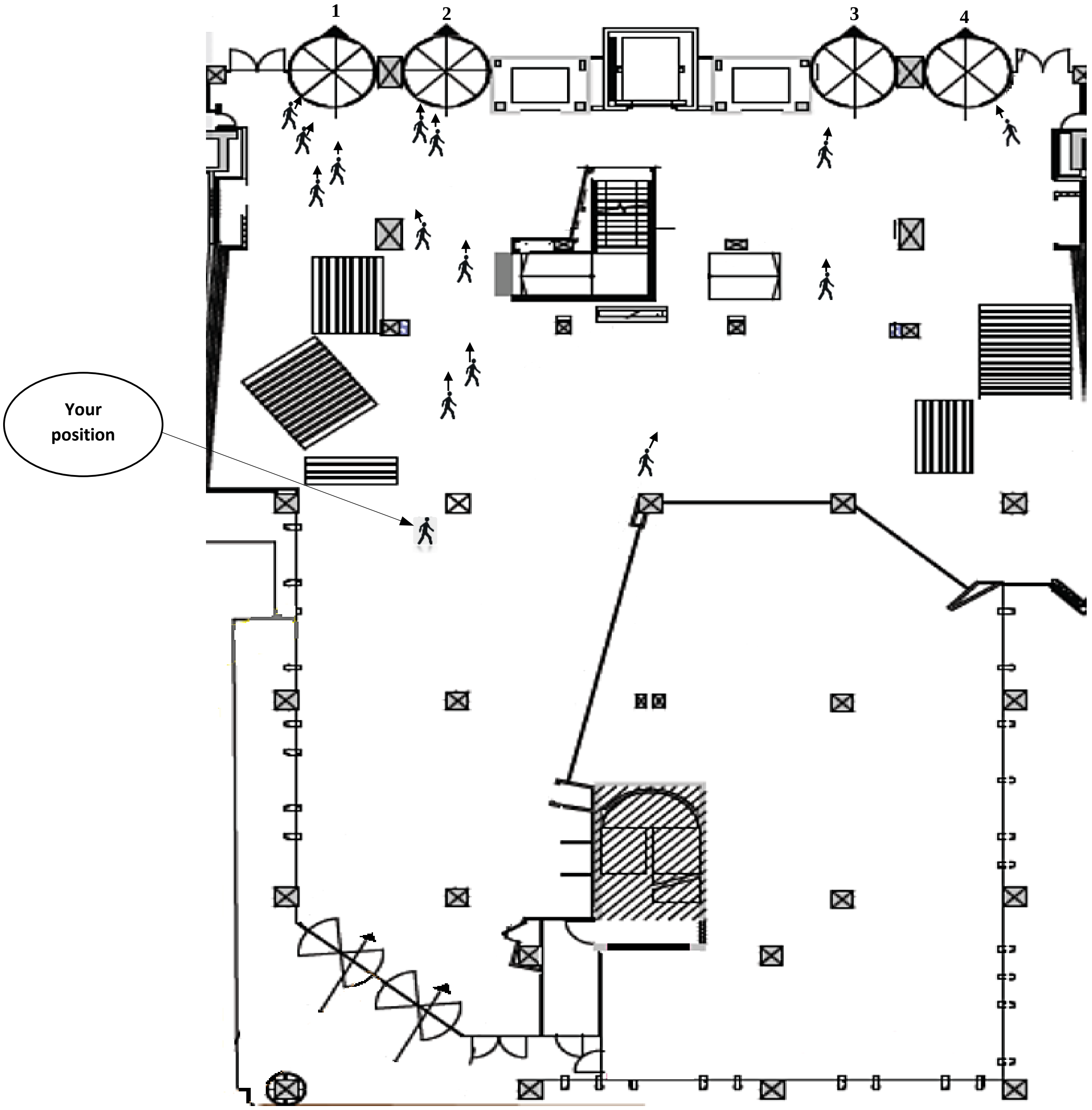

Your Choice:

Exit 1   Exit 2   Exit 3   Exit 4

Scenario 7

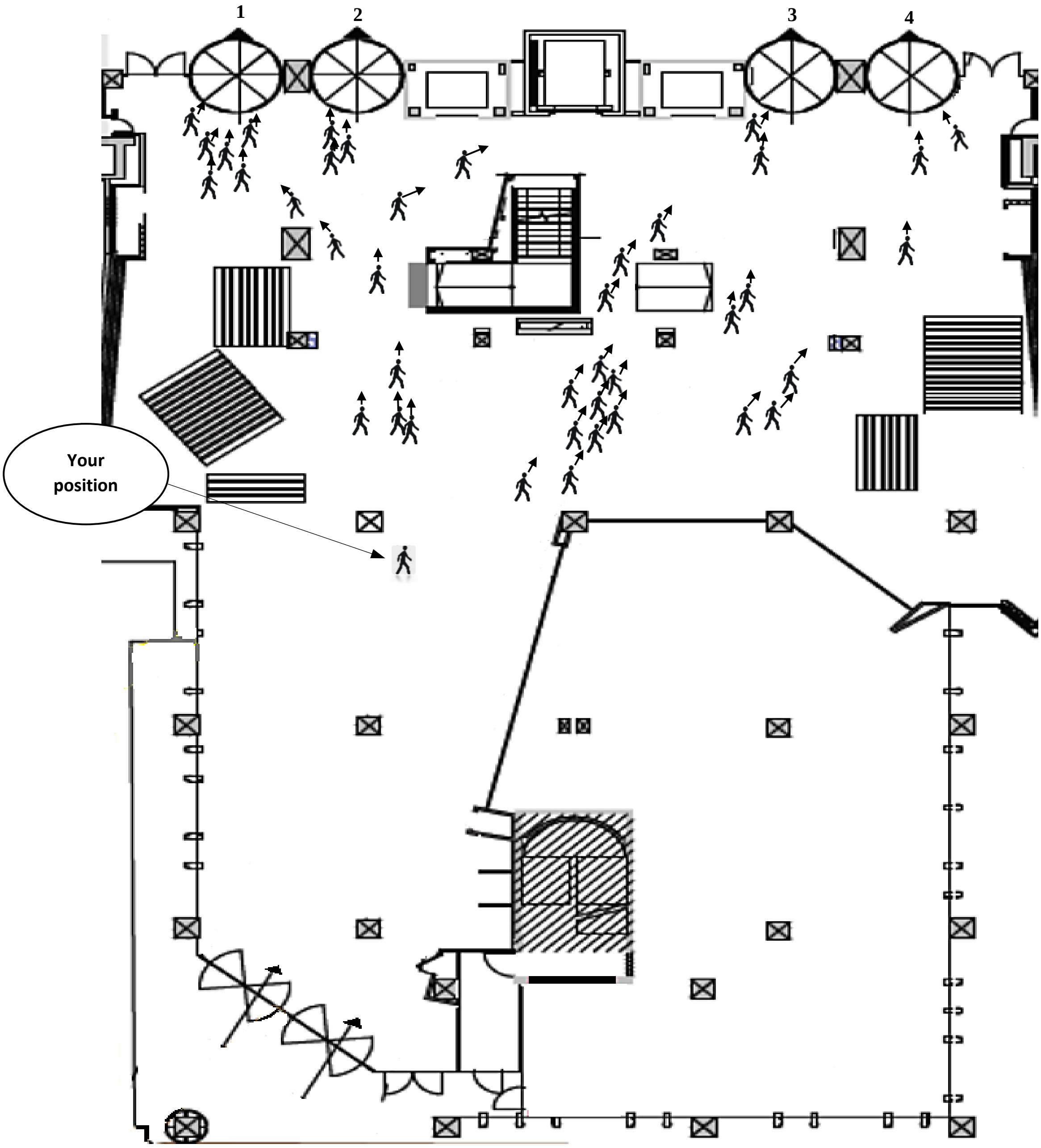

Your Choice:

Exit 1   Exit 2   Exit 3   Exit 4

Scenario 8

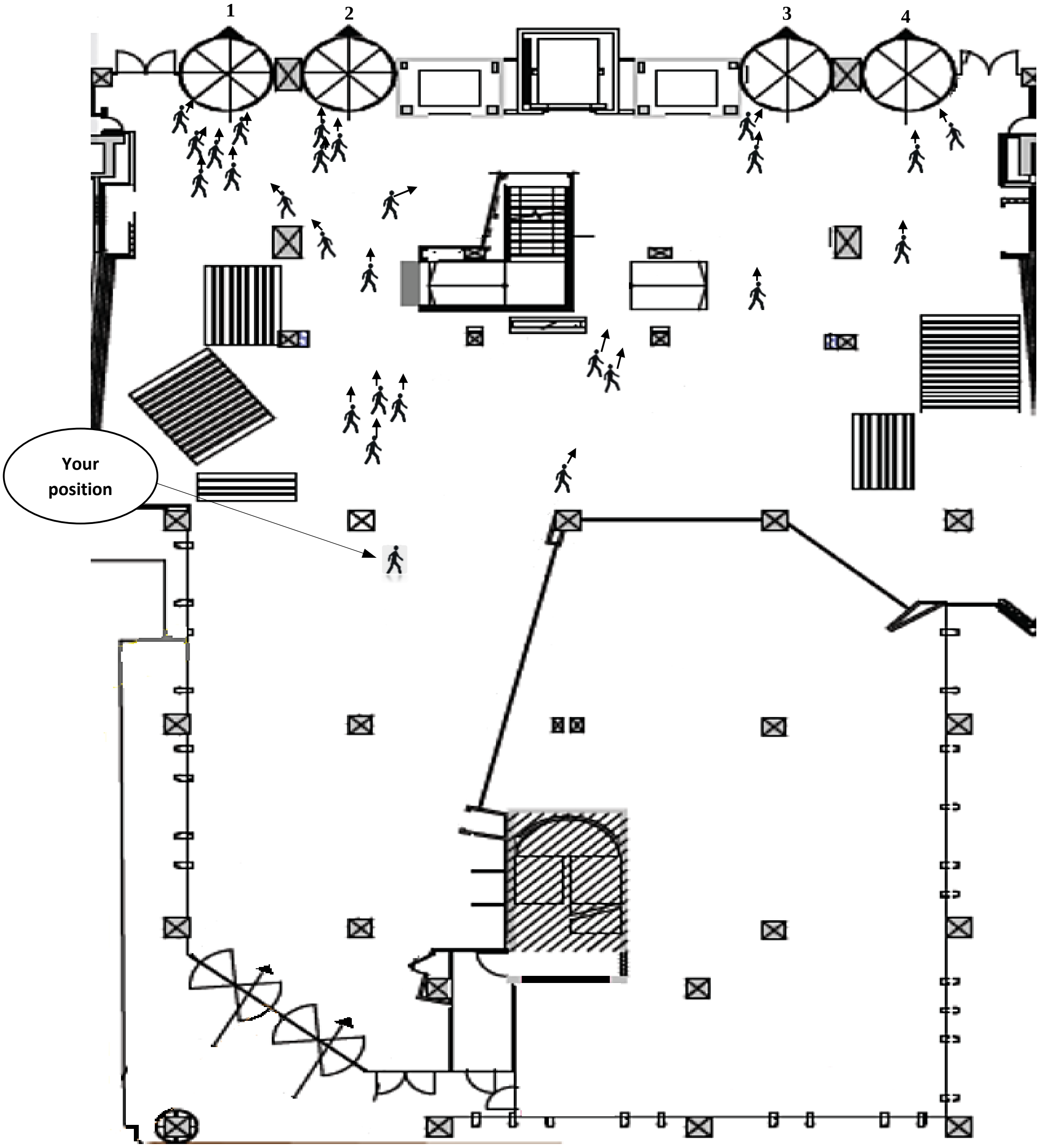

Your Choice:

Exit 1 Exit 2 Exit 3 Exit 4

Scenario 9

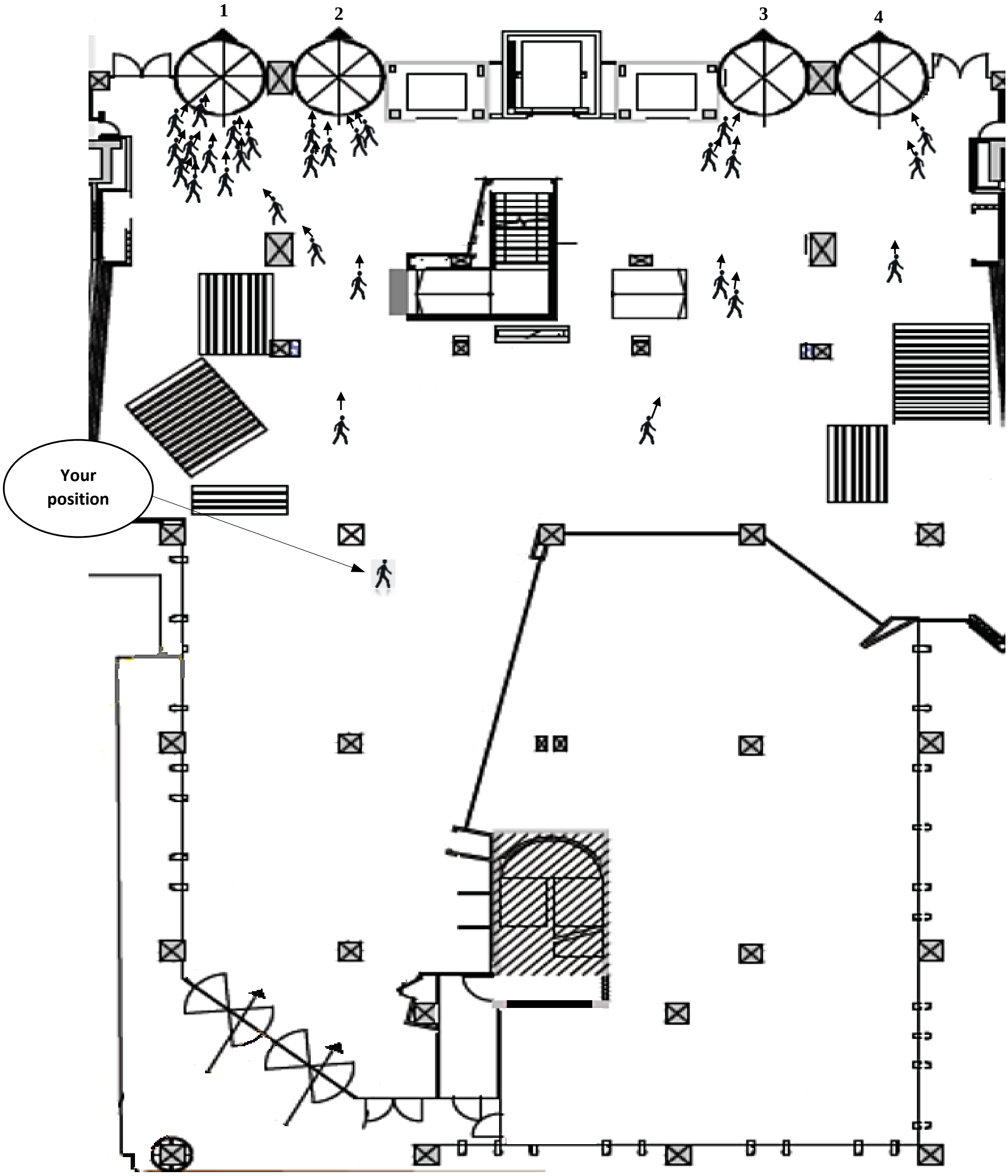

Your Choice:

Exit 1   Exit 2   Exit 3   Exit 4

Scenario 10

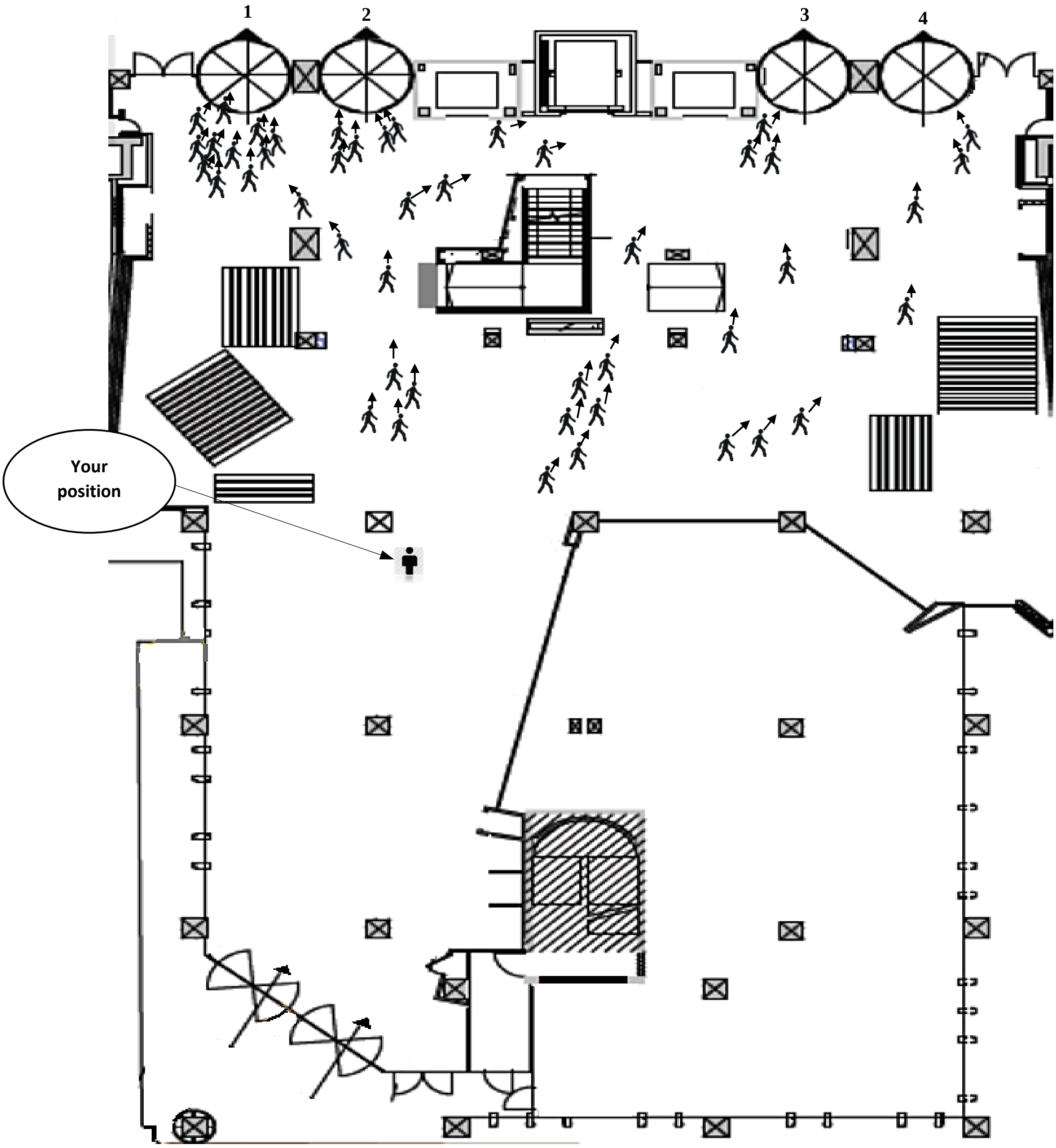

Your Choice:

Exit 1 | Exit 2 | Exit 3 | Exit 4

Scenario 11

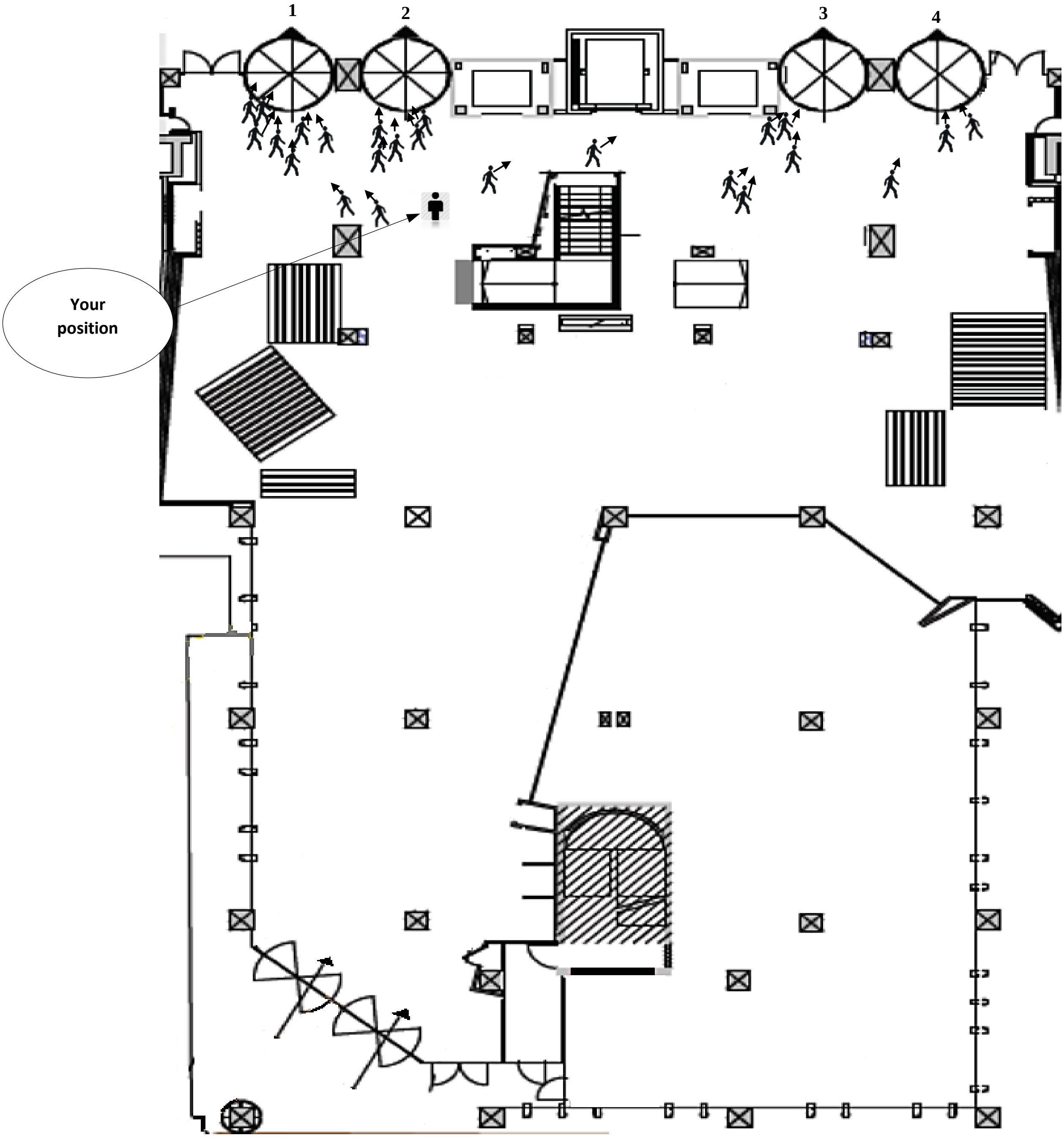

Your Choice:

Exit 1 Exit 2 Exit 3 Exit 4

Scenario 12

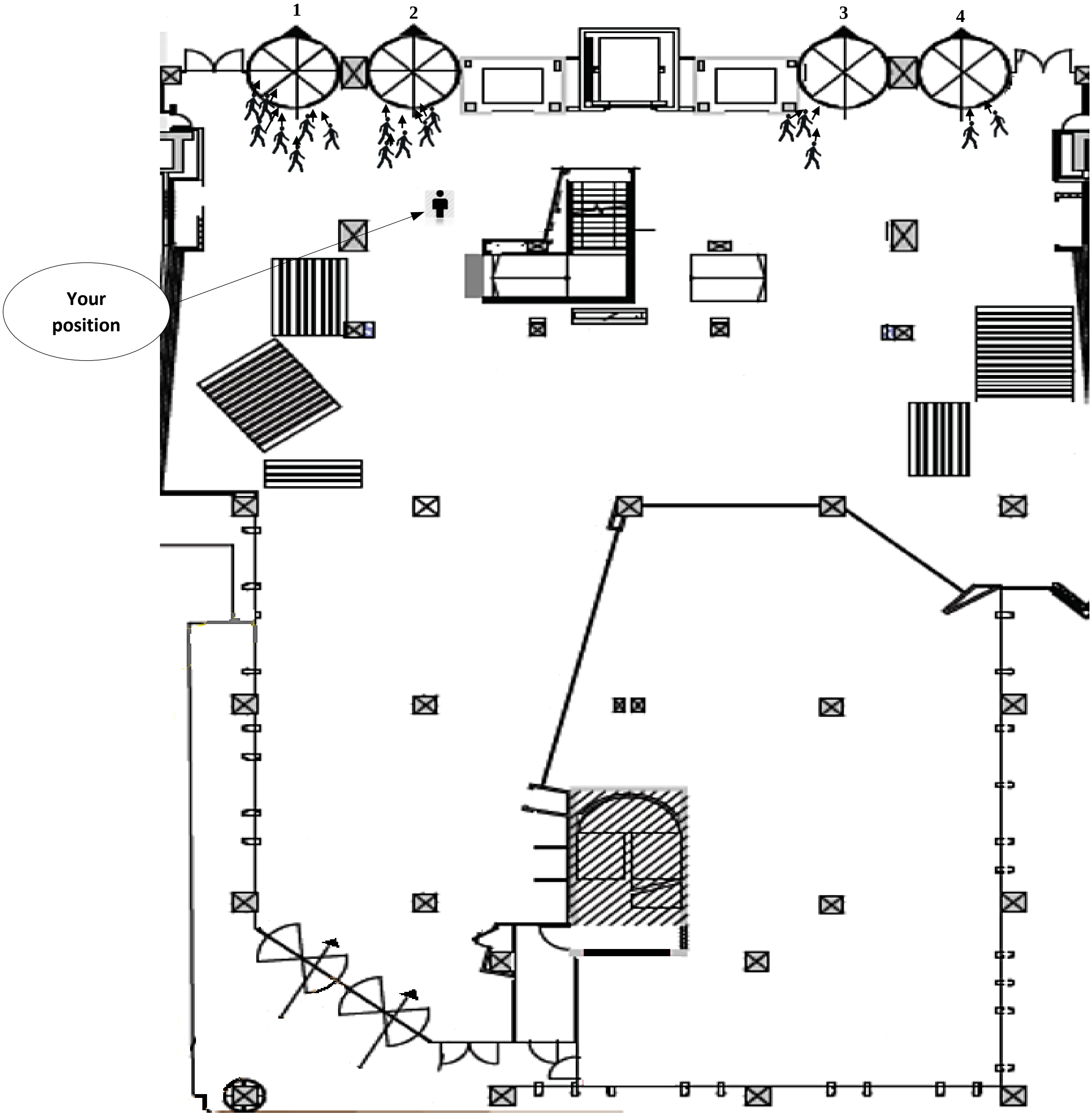

Your Choice:

Exit 1 Exit 2 Exit 3 Exit 4

Scenario 13

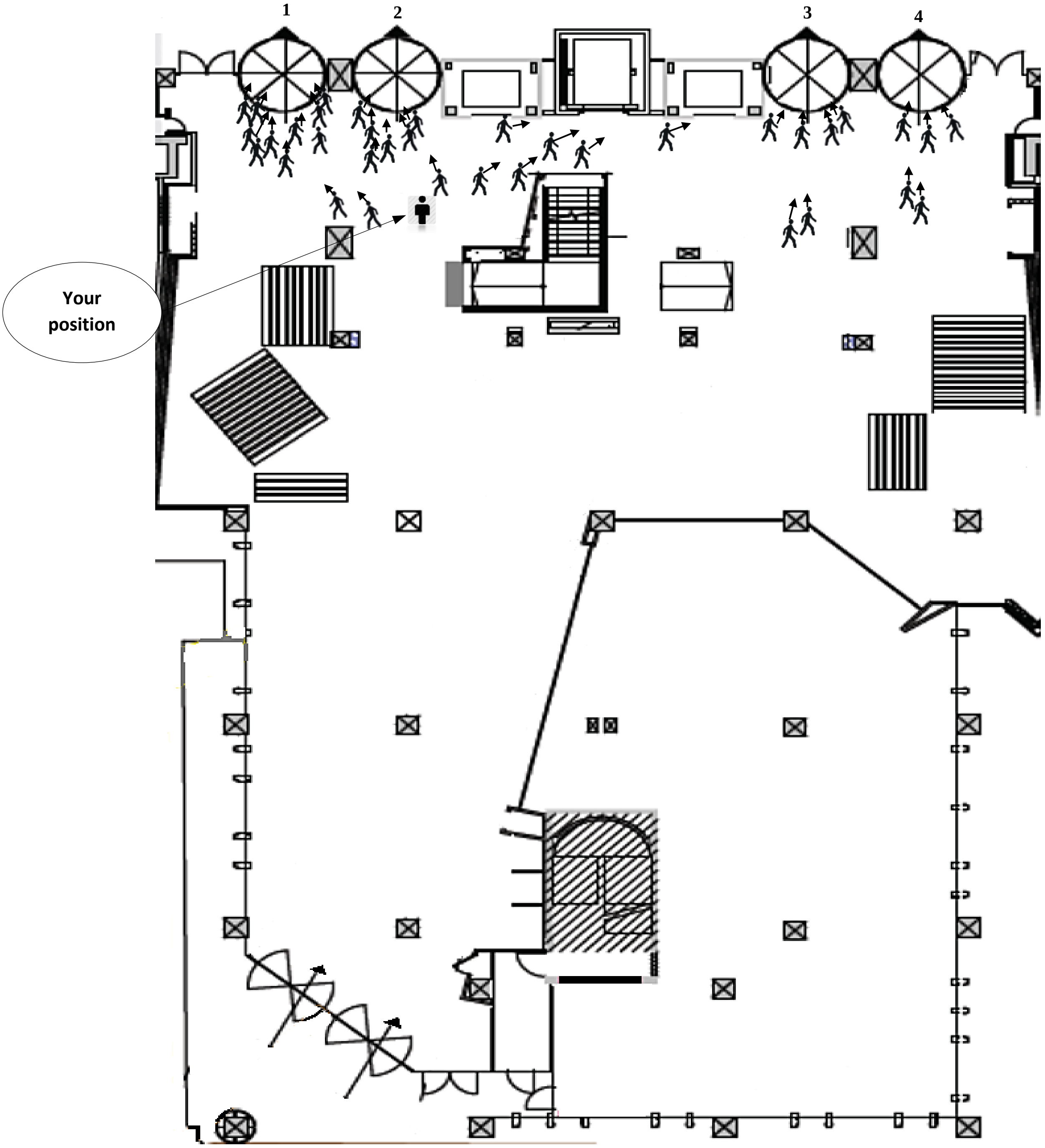

Your Choice:

Exit 1 Exit 2 Exit 3 Exit 4

Scenario 14

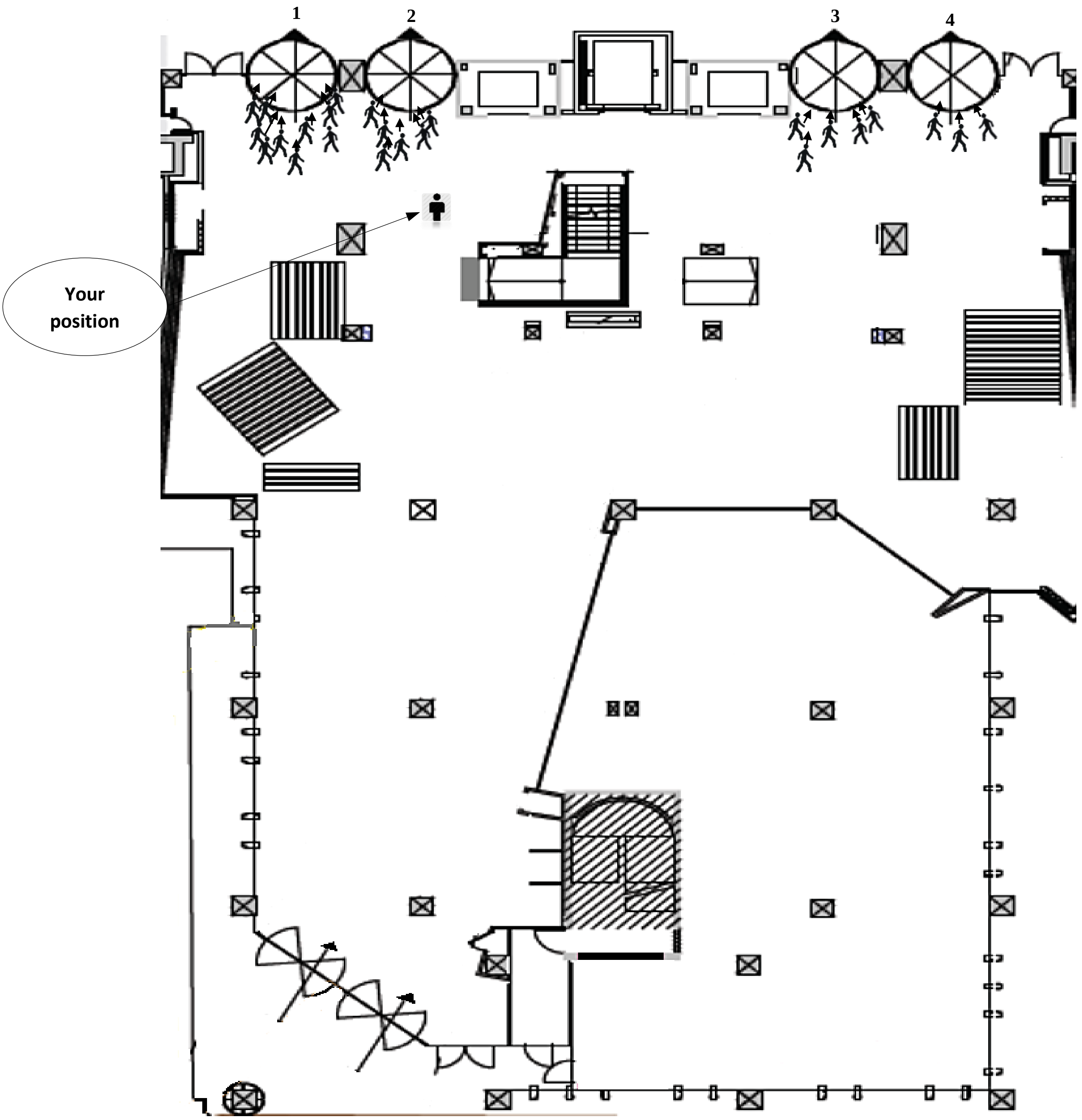

Your Choice:

Exit 1 Exit 2 Exit 3 Exit 4

SC (I) scenarios - Block (II)

Scenario 1

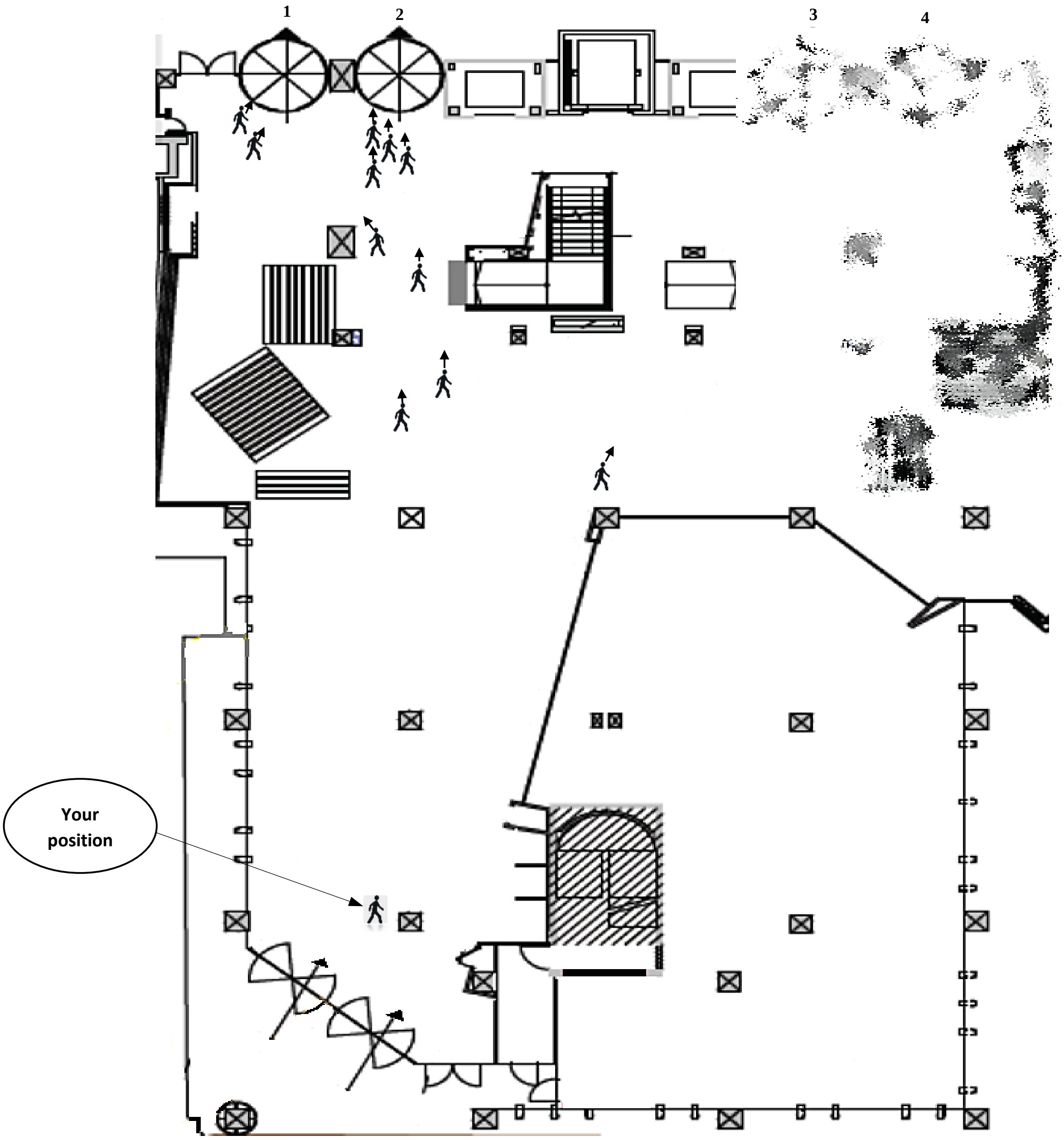

Your Choice:

Exit 1 Exit 2 Exit 3 Exit 4

Scenario 2

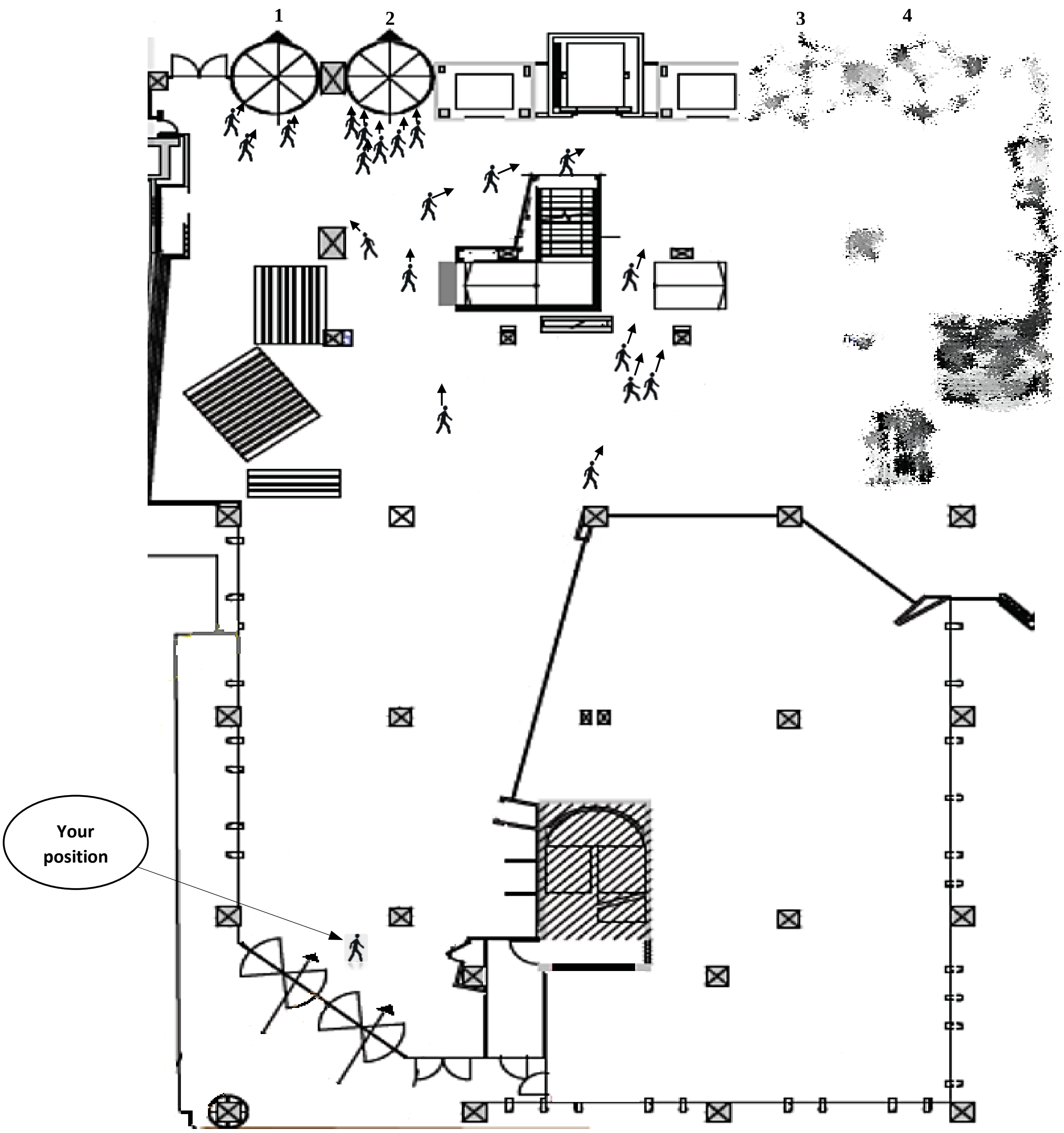

Your Choice:

Exit 1

Exit 2

Exit 3

Exit 4

Scenario 3

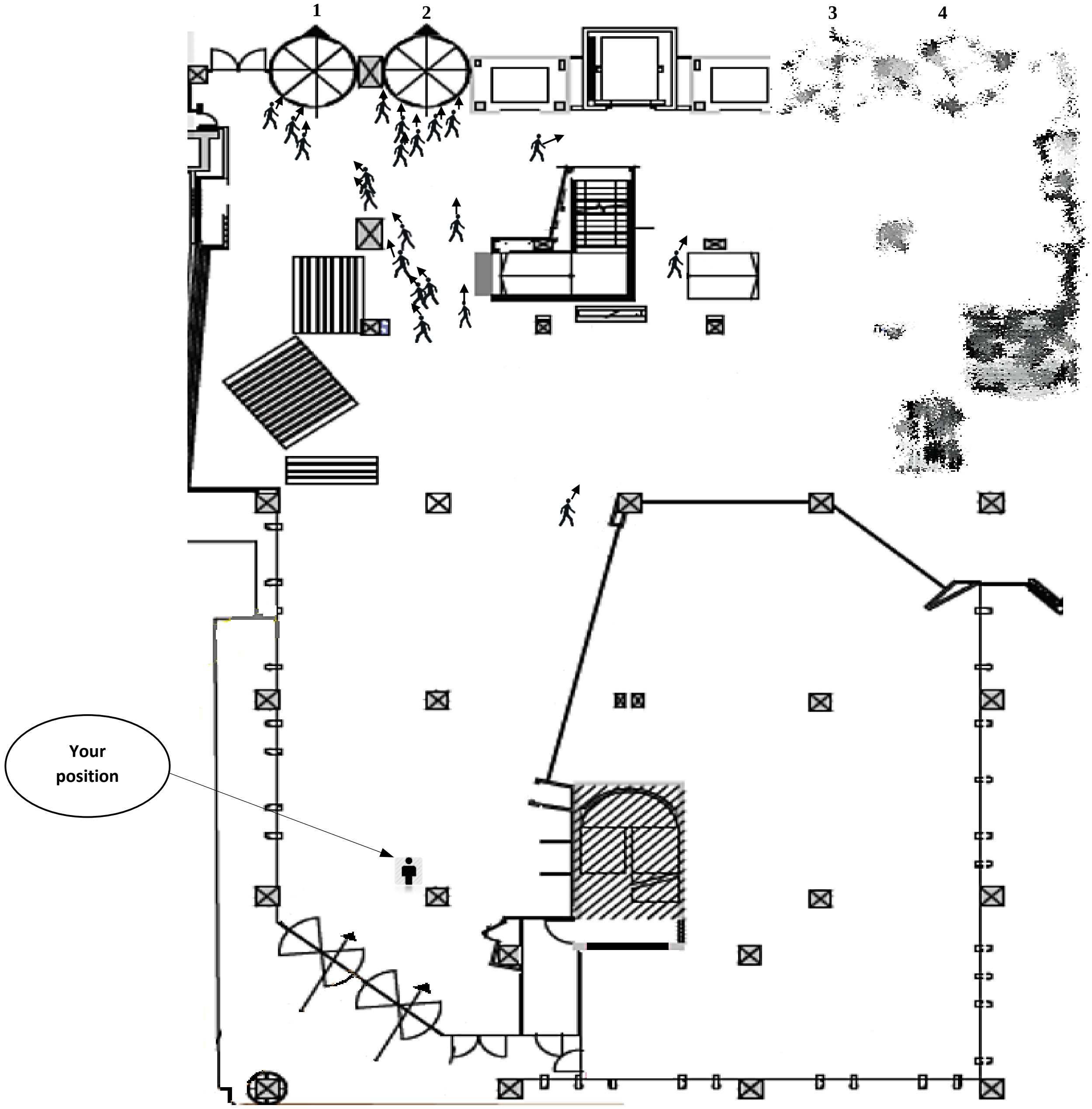

Your Choice:

Exit 1 Exit 2 Exit 3 Exit 4

Scenario 4

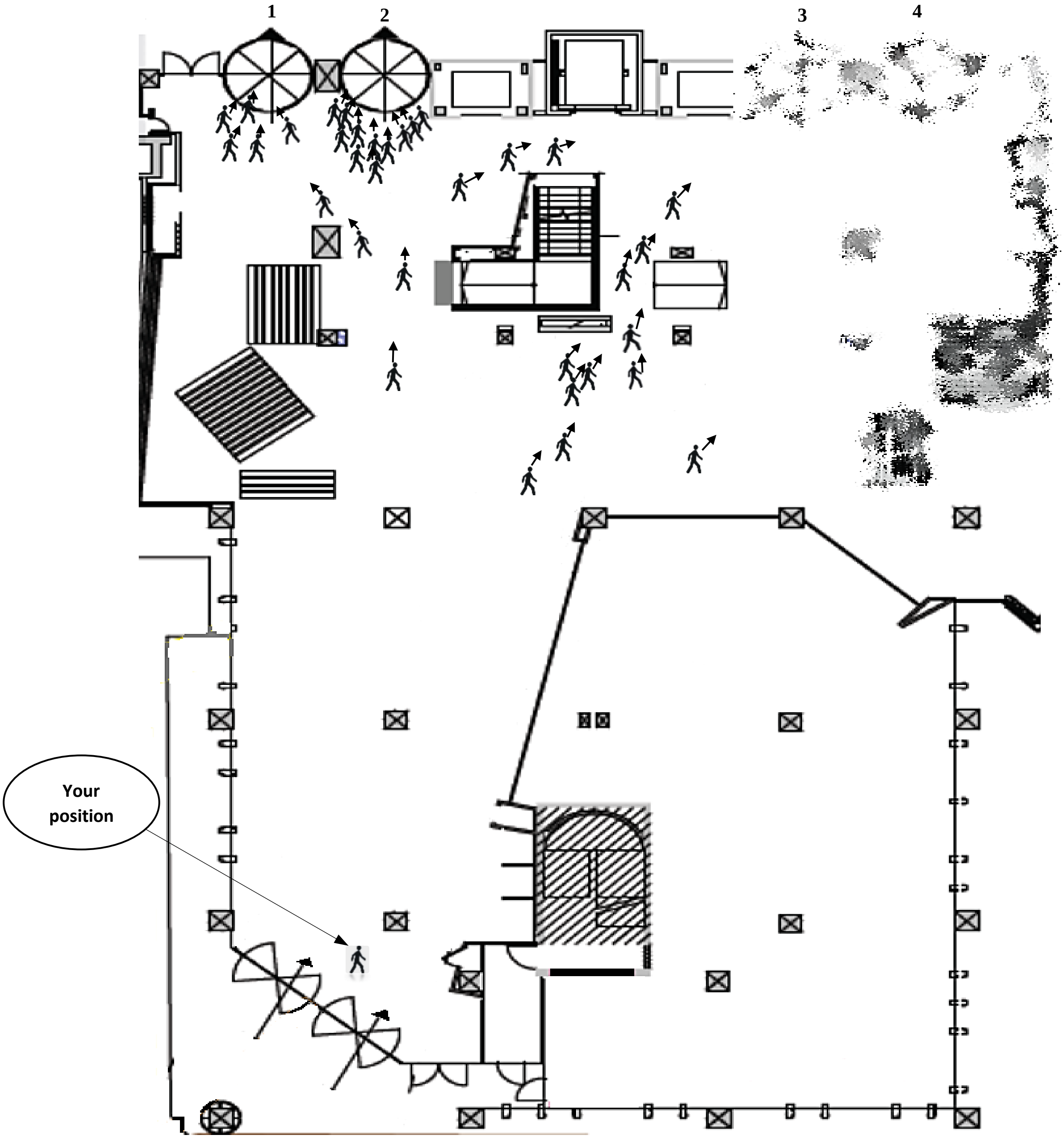

Your Choice:

Exit 1 Exit 2 Exit 3 Exit 4

Scenario 5

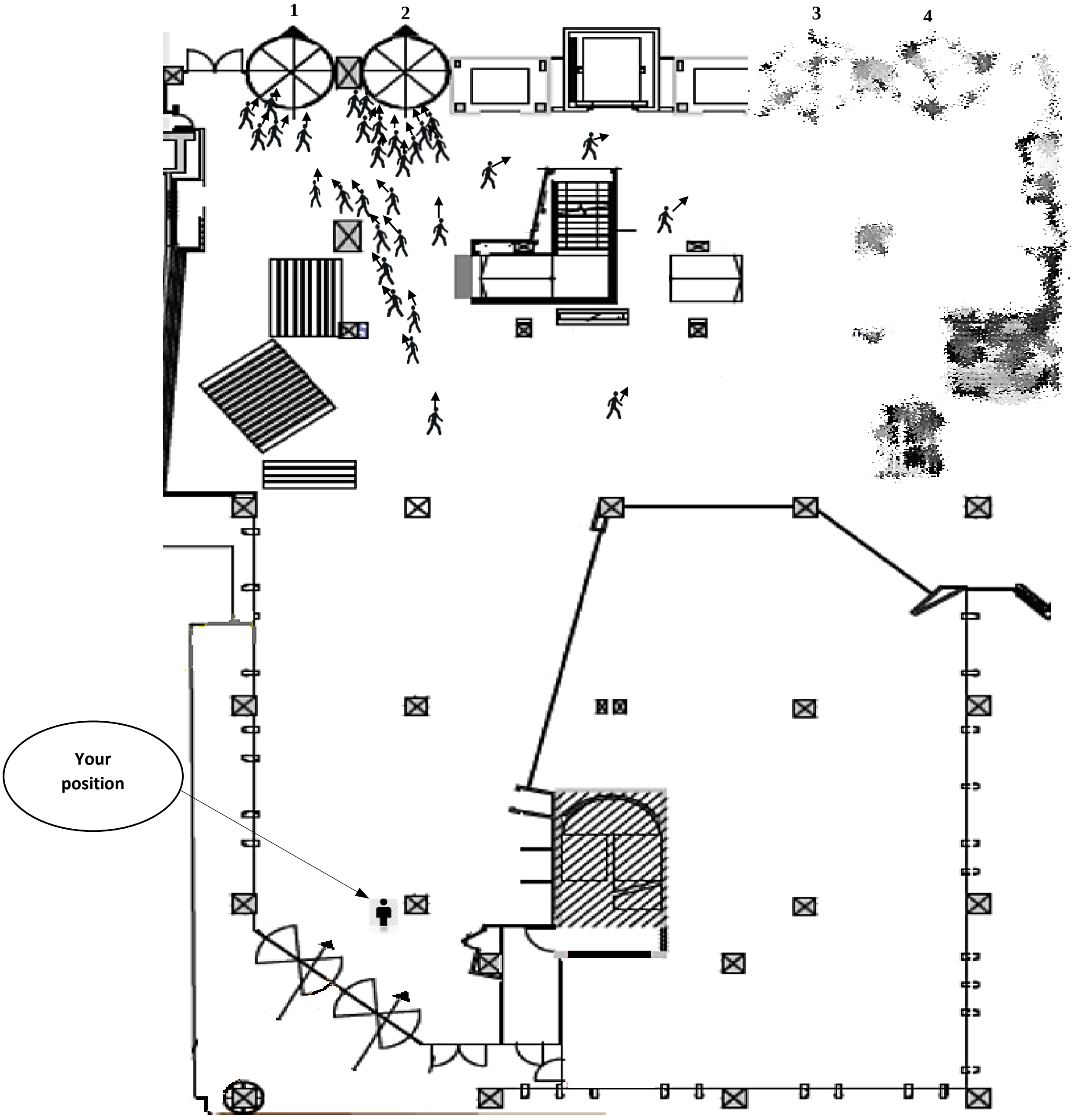

Your Choice:

Exit 1   Exit 2   Exit 3   Exit 4

Scenario 6

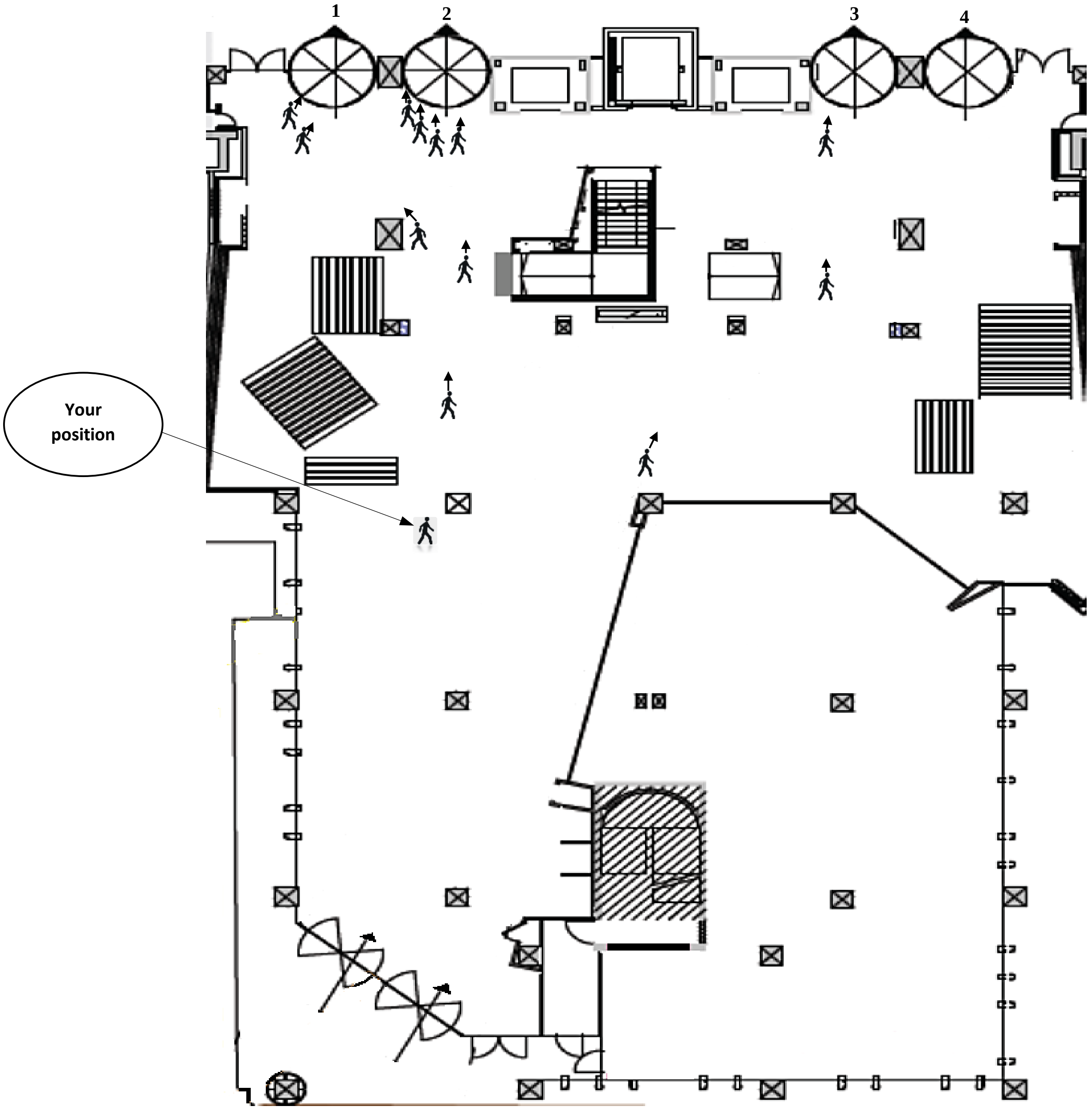

Your Choice:

Exit 1 Exit 2 Exit 3 Exit 4

Scenario 7

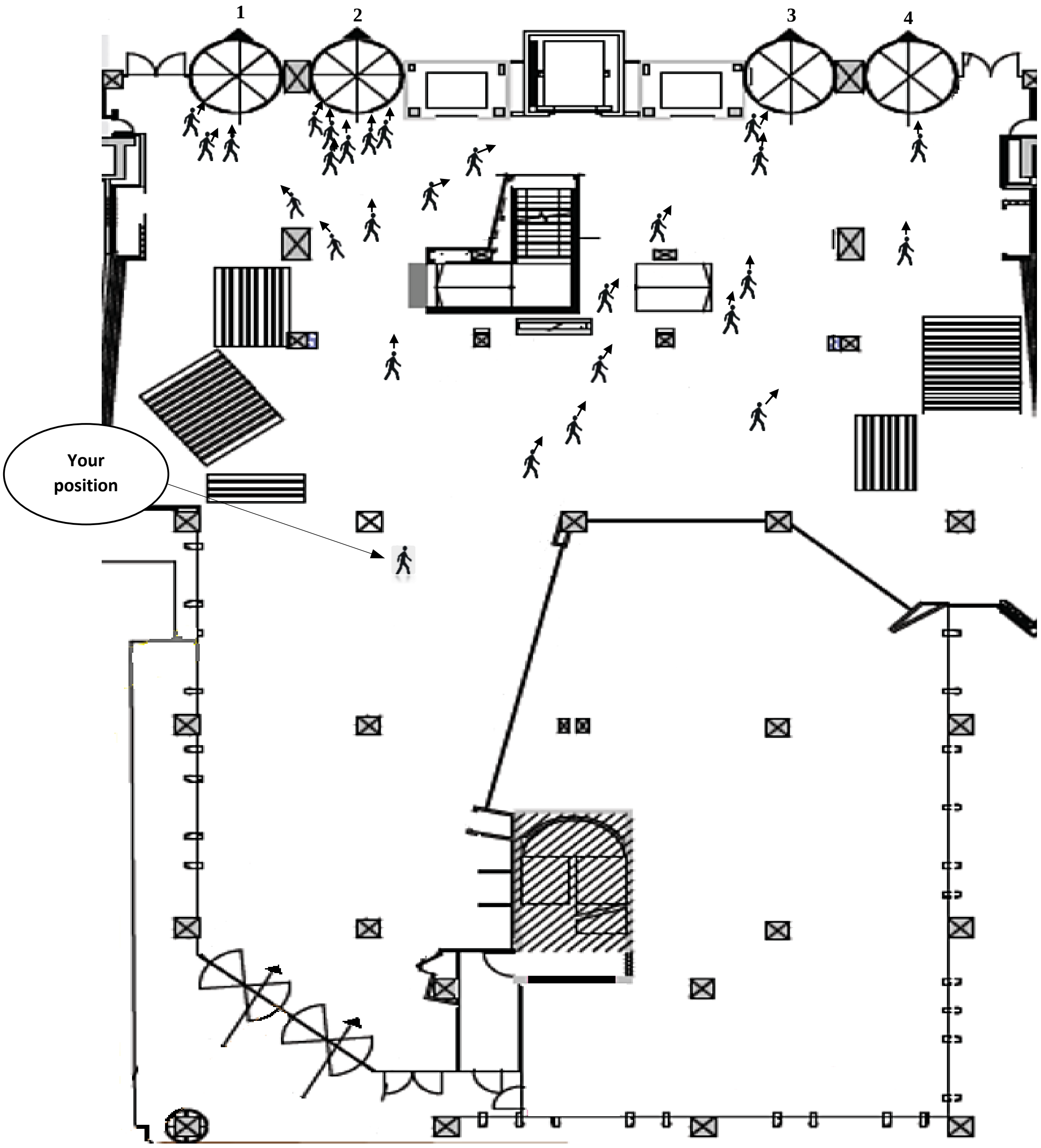

Your Choice:

Exit 1 Exit 2 Exit 3 Exit 4

Scenario 8

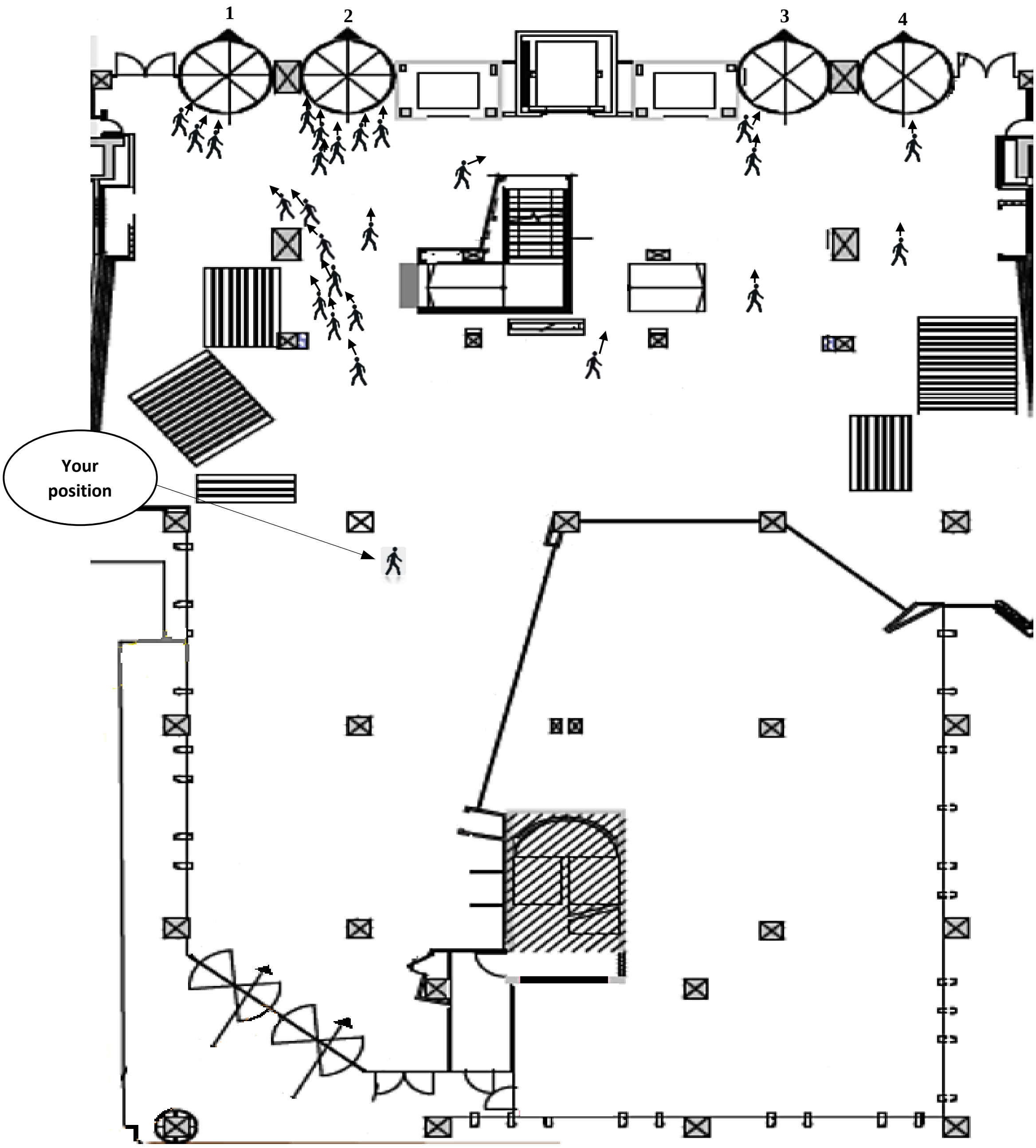

Your Choice:

Exit 1 Exit 2 Exit 3 Exit 4

Scenario 9

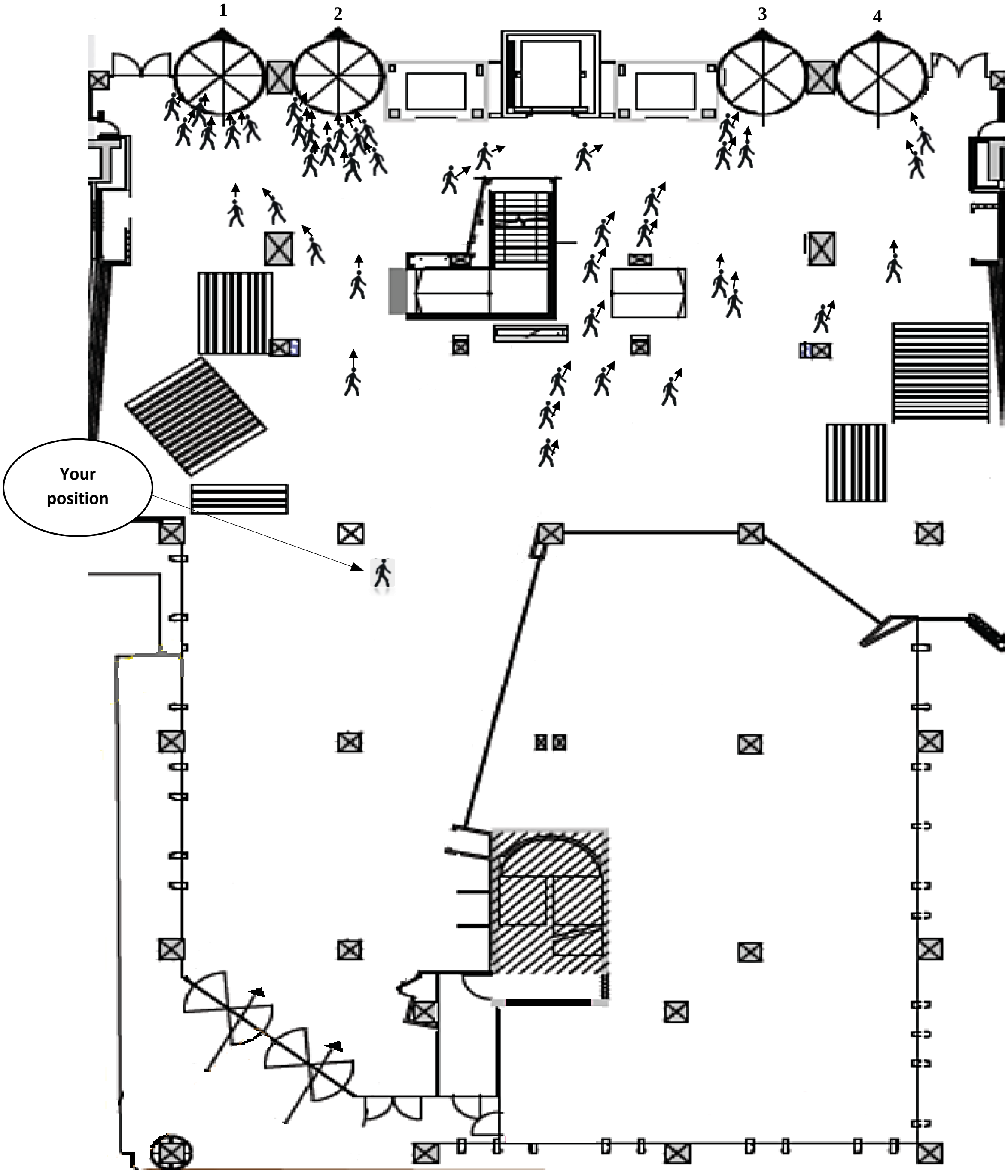

Your Choice:

Exit 1

Exit 2

Exit 3

Exit 4

Scenario 10

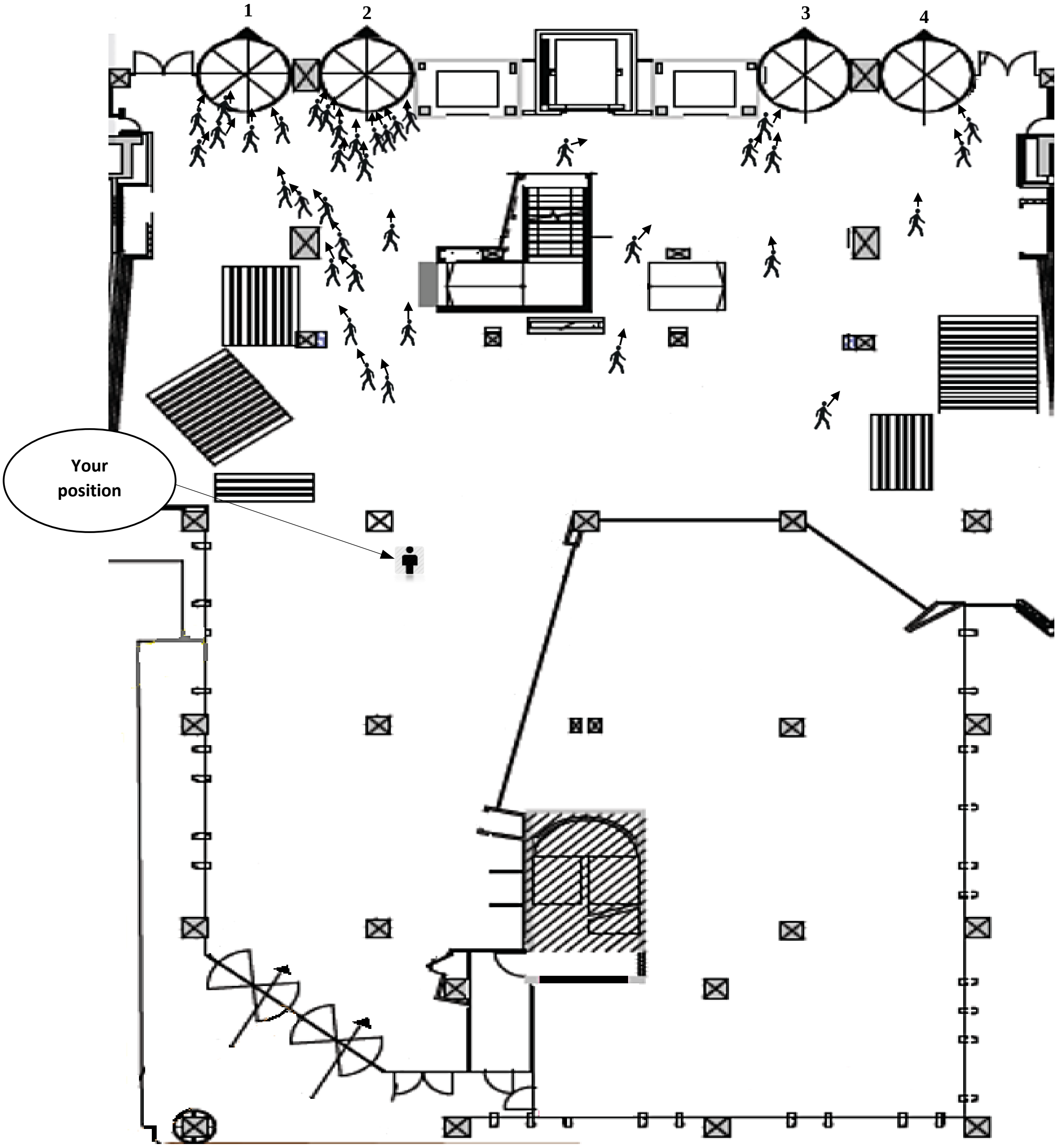

Your Choice:

Exit 1 Exit 2 Exit 3 Exit 4

Scenario 11

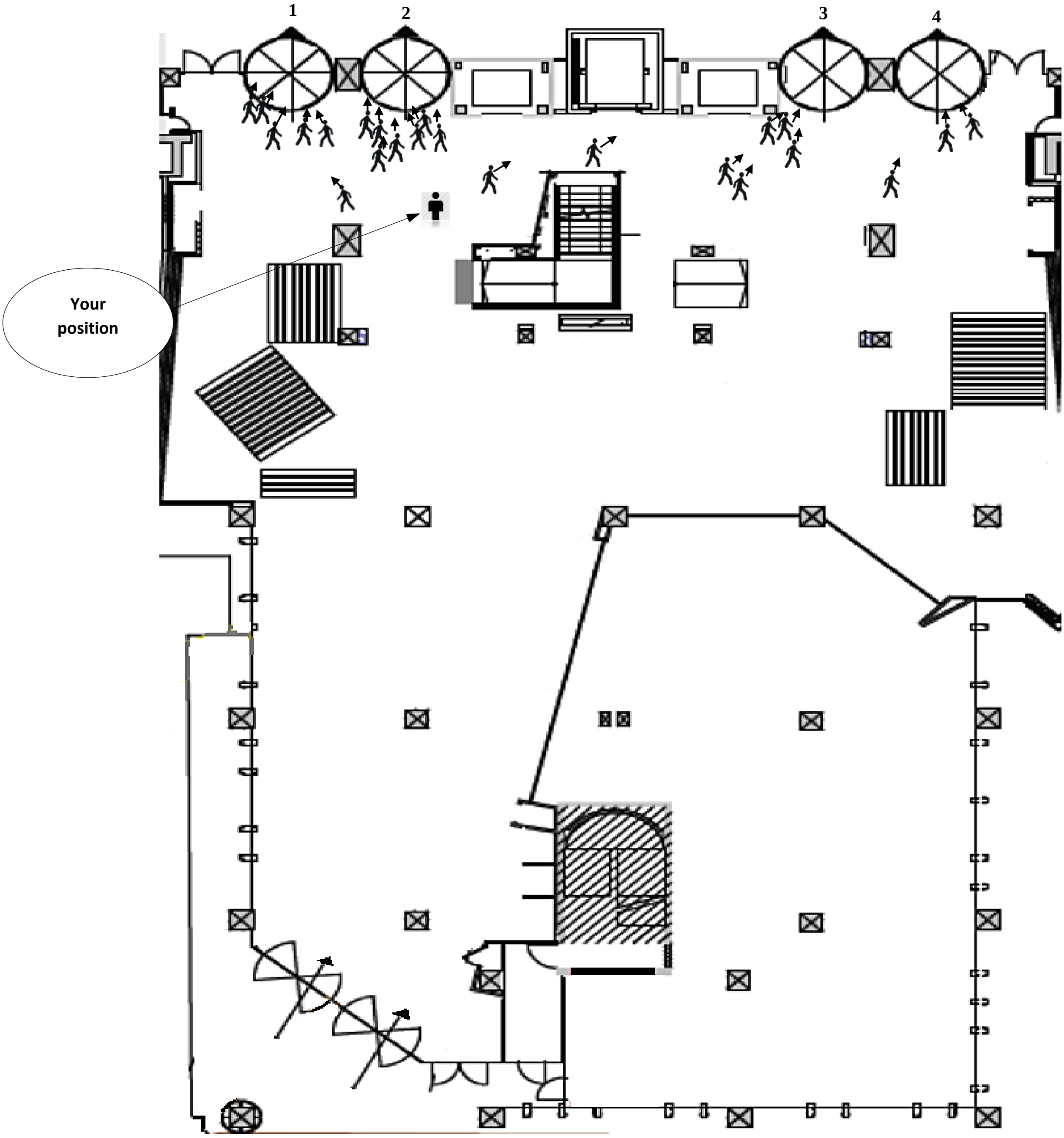

Your Choice:

Exit 1 Exit 2 Exit 3 Exit 4

Scenario 12

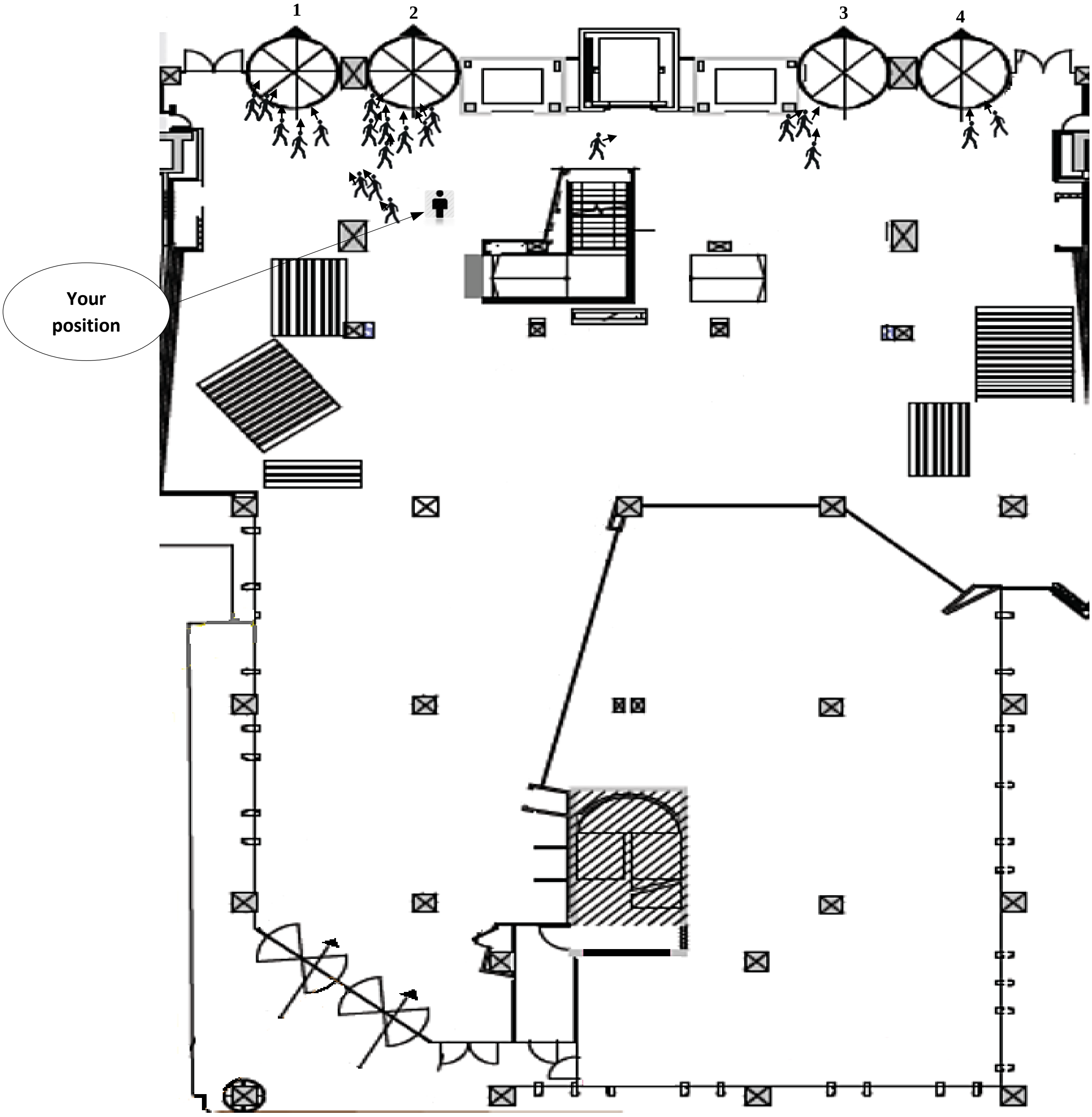

Your Choice:

Exit 1 Exit 2 Exit 3 Exit 4

Scenario 13

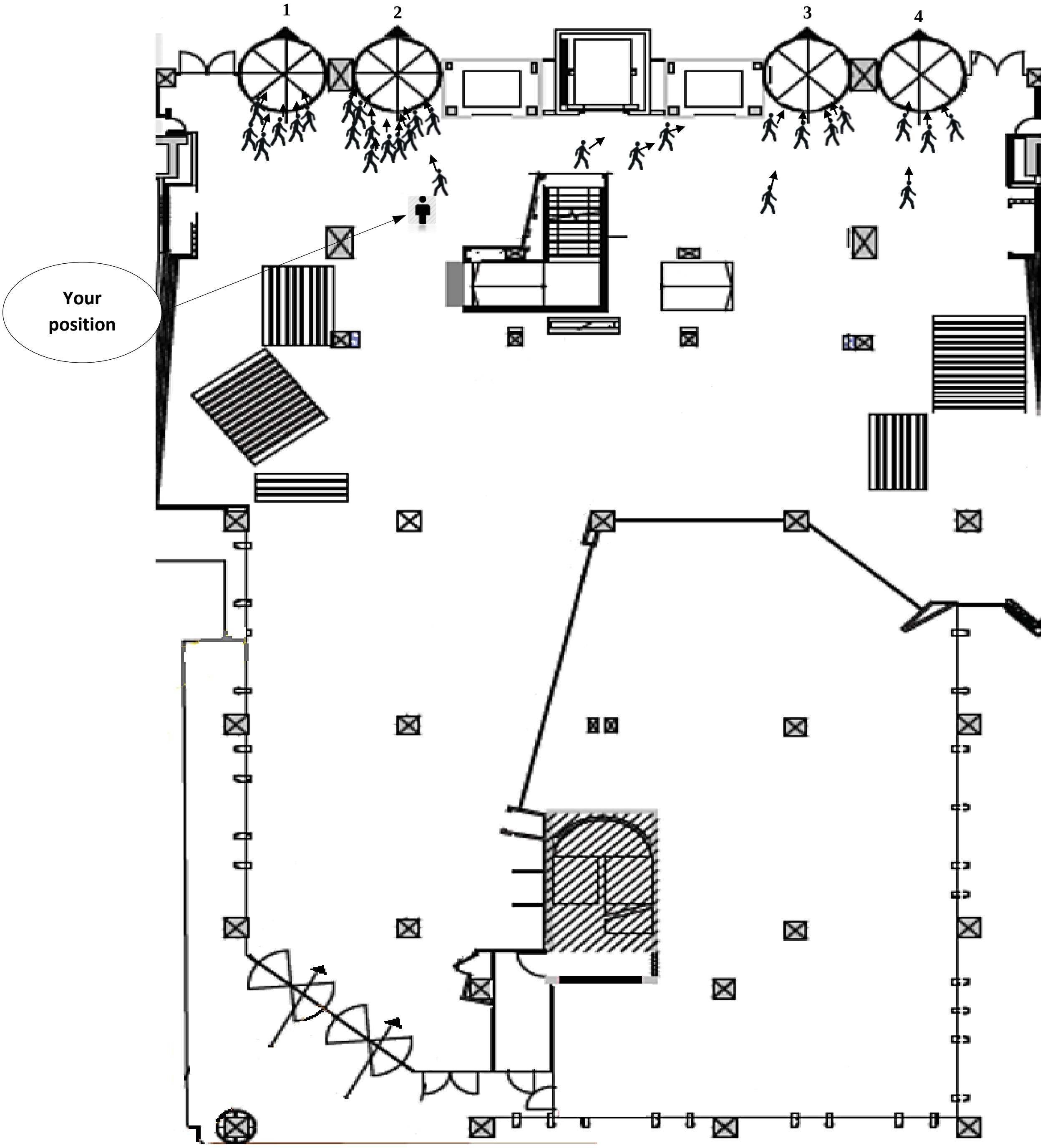

Your Choice:

Exit 1 | Exit 2 | Exit 3 | Exit 4

Scenario 14

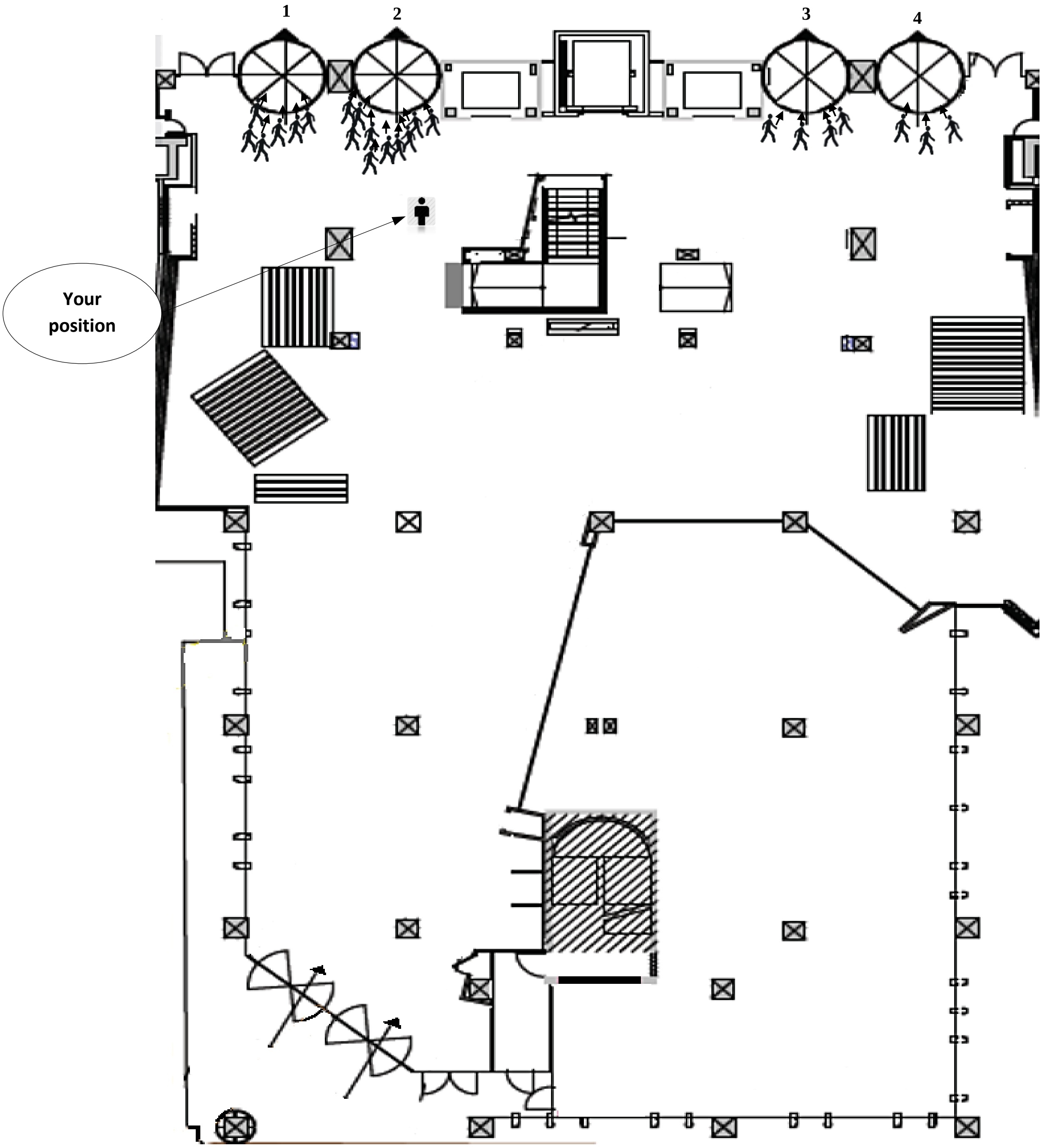

Your Choice:

Exit 1 Exit 2 Exit 3 Exit 4
