# Supplementary material for: How Simple Hypothetical-Choice Experiments Can Be Utilized to Learn Humans’ Navigational Escape Decisions in Emergencies
Source: PLoS One. 2016 Nov 21;11(11):e0166908. doi: 10.1371/journal.pone.0166908 (PMC5117746; doi:10.1371/journal.pone.0166908)
Supplement: S2 Fig — (PDF) [file pone.0166908.s002.pdf]

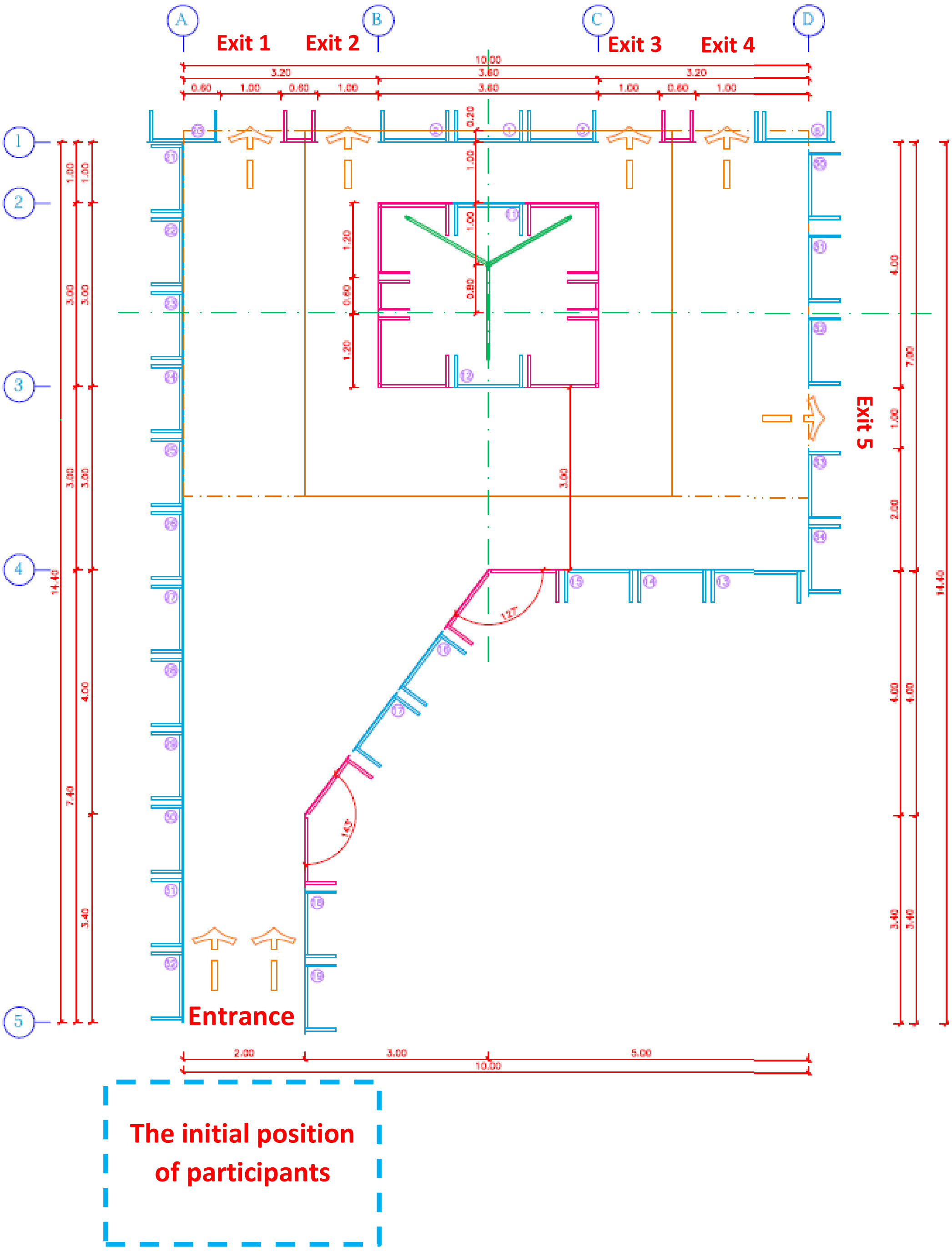

The general map of the evacuation room built for mock evacuation experiments. In total, 5 exits were built, but for each trial run only a subset of them were available to the participants. The location of the tripod legs used for mounting the camera has also been shown in green line inside the obstacle area.
