# Supplementary material for: How Simple Hypothetical-Choice Experiments Can Be Utilized to Learn Humans’ Navigational Escape Decisions in Emergencies
Source: PLoS One. 2016 Nov 21;11(11):e0166908. doi: 10.1371/journal.pone.0166908 (PMC5117746; doi:10.1371/journal.pone.0166908)
Supplement: S2 Table — (PDF) [file pone.0166908.s004.pdf]

As mentioned in the main manuscript, The trials in total consisted of 20 scenario runs. Each scenario is a combination of certain design factors including (1) the number of evacuees (either 75 or 150), (2) the number of available exits (2, 3 or 4) and (3) their spatial distribution as well as (4) the widths of the exits (50 cm or 100 cm). The details of these combinations are provided in Table S1 below. Figure S1 shows the map based on which we built the evacuation environment.

Details of the trial runs of the realistic choice experiments.

**Scenario number**	**Number of evacuees**	**Available exits**	**Exit widths**
1	75	1,2,3,4	w1=w2=w3=w4=100 cm
2	75	1,2,3,4	w1=w2=w3=w4=100 cm
3	75	1,3,4	w1=w3=w4=100 cm
4	75	1,3,4	w1=w3=w4=100 cm
5	75	1,2,4	w1=w2=w4=100 cm
6	75	1,2,4	w1=w2=w4=100 cm
7	150	1,2,3,4	w1=w2=w3=w4=100 cm
8	150	1,2,3,4	w1=w2=w3=w4=100 cm
9	150	1,2,3,4	w1=w2=w3=w4=50 cm
10	150	1,2,3,4	w1=w2=w3=w4=50 cm
11	150	1,3,4	w1=w3=w4=50 cm
12	150	1,2,3	w1=w2=w3=50 cm
13	150	2,3	w2=w3=50 cm
14	150	1,4	w1=w4=50 cm
15	150	2,5	w2=w5=50 cm
16	150	2,5	w2=w5=50 cm
17	150	2,5	w2=50 cm, w5= 100 cm
18	150	2,5	w2=100 cm, w5=50 cm
19	150	2,3,5	w2=w3=w5=50 cm
20	150	2,3,5	w2=w5=50 cm, w3=100 cm
