# Supplementary material for: How Simple Hypothetical-Choice Experiments Can Be Utilized to Learn Humans’ Navigational Escape Decisions in Emergencies
Source: PLoS One. 2016 Nov 21;11(11):e0166908. doi: 10.1371/journal.pone.0166908 (PMC5117746; doi:10.1371/journal.pone.0166908)
Supplement: S1 Text — (DOCX) [file pone.0166908.s006.docx]

**Extraction of Trajectories**

To extract the trajectory of each individual person, the software PeTrack was used. A camera GoPro Hero4 was located at 8 m height above the floor recording the scene nearly perpendicular to the ground. A GoPro has been chosen, because of its wide angle lens (124° by 93° to the center of the image border) and the possibility to record high resolution images with a high frame rate. Snapshots of the raw footage can be seen in Fig. 4 The recordings consist of progressive images with squared pixel, a resolution of 1920 pixel by 1440 pixel and a frame rate of 50 frames per second. The high frame rate was required because of experiments where the participants were allowed to run. The frame rate of 50 f/s allows a spatial resolution of 0.1 meter per frame for the trajectories assuming a human speed of 5 m/s. Single resulting trajectories can be seen in Figs. 5 and 6. The perpendicular view was chosen to minimize the occlusion of the participants among each other. Caused especially by the wide angle lens, the image distortion was large. The parameters are fitted by equating pixel positions with distorted real world positions of characteristic points of a picture sequence with a pattern. The quality of the un-distortion can be seen in Fig. 1, by means of a grid overlay with a resolution of 1 m by 1 m on ground level.


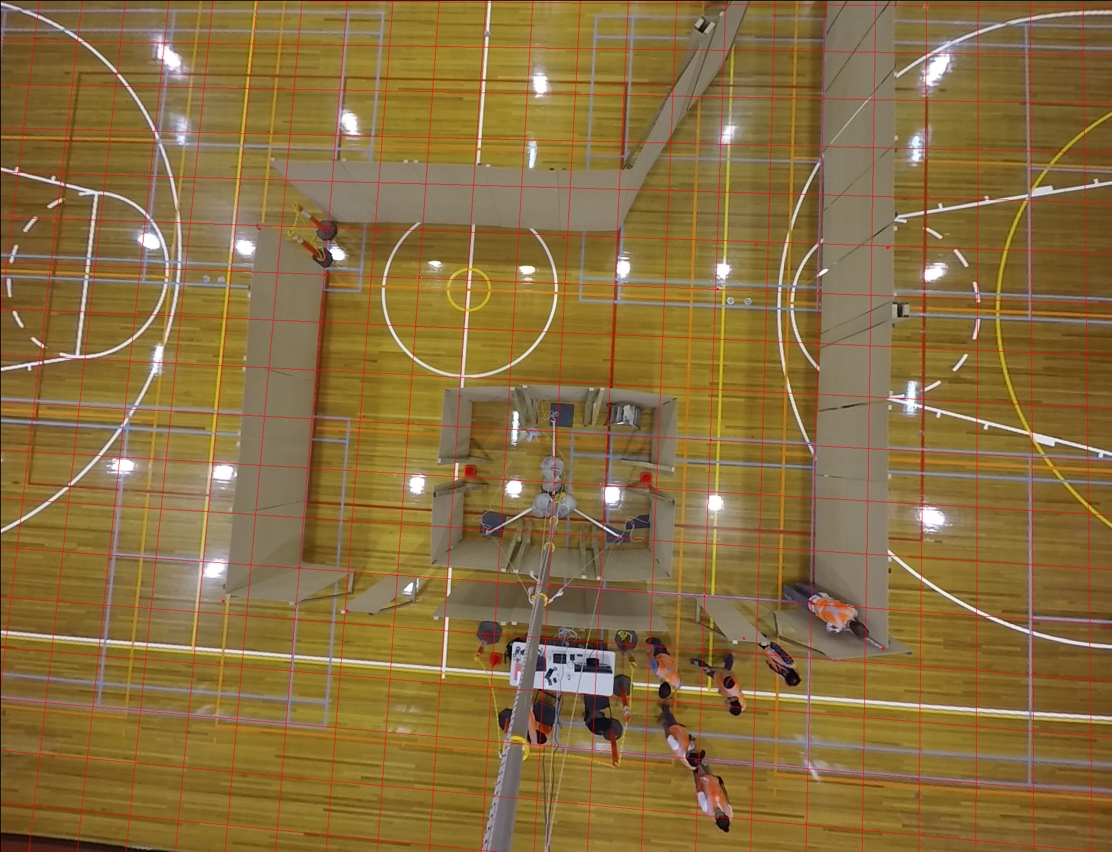


**Fig. 1.** Single image of the recordings after un-distortion. The red grid overlay with a resolution of 1 m by 1 m on ground level shows the quality of the correction by parallel lines to the printed pattern on the ground.

For the mapping of the real world coordinate system to the image coordinate system an iterative method based on Levenberg-Marquardt optimization was used, which minimizes the re-projection error using the sum of squared distances between the observed projections of image points and the projected points of real measurement points. The measurement points in the observation area have been captured on the ground and in head height using a leveling pole. Fig. 2 shows 22 points, where the centers of the red circles are the observed projections of the image points and the blue dots are the projected points of the measurement points. The average pixel error is 1.7 pixel (maximum of 4.1 pixel) in image space and the average error is 0.015 m (maximum of 0.036 m) in the real world in head height. These errors include the error due to the distortion and the inaccuracy of the measurement. The resulting 3D coordinate system is drawn in Fig. 2 with axes of 5 m length.


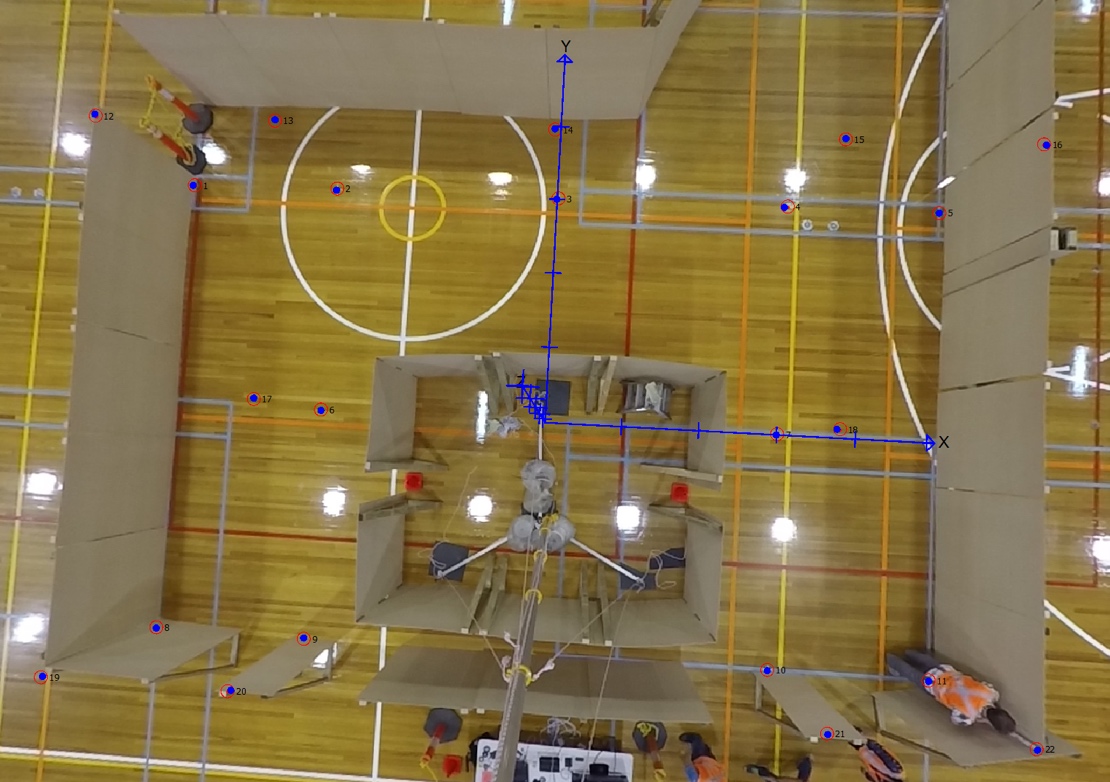


**Fig. 2.** For the mapping of the real world coordinate system to the image coordinate system measurement points have been captured in the observation area on the ground and in head height using a leveling pole. Here 22 points were used, where the centers of the red circles are the observed projections of the image points and the blue dots are the projected points of the measurement points. The resulting 3D coordinate system is drawn with axes of 5 m length.

For the recognition of the participants we asked them to wear colored beanies to facilitate the automatic recognition. The head was marked, because the marking enables robust detection and this part of the body is the only one, which can be seen in dense crowds without occlusion from the chosen point of view. The color green was selected, because this color did not occur in the observation area. It was ensured that the people did not wear clothes with this color. Every pixel in a subspace of the HSV color space was supposed to belong to the beanie. For a robust detection the HSV color space has been used, because the hue channel interval could be chosen small but the value interval could be chosen large according to variable shadings caused by different angles of light incidence. Small holes and branches inside the selection mask were eliminated by morphological operations (open and close with a kernel size of 15% of the head diameter). Pixels of the selection mask not belonging to the hat are shaded in Fig. 3 so that only greenish pixels are bright. Connected components inside the selection mask with a size larger than 50% and smaller than 150% of the average size of a hat were approximated by ellipses. The center of the ellipses identifies the position of a person, if the aspect ratio of the axes is smaller than 2. The perspective distortion leads to an error, because the hat of a person is seen more from the side turned to the camera. Thus the assumed position is slightly shifted to the position perpendicular to the camera. With an optimal hat shape of a hemisphere the maximum error would be 0.056 m for the maximum angel of view of 47° for the chosen experimental setup.


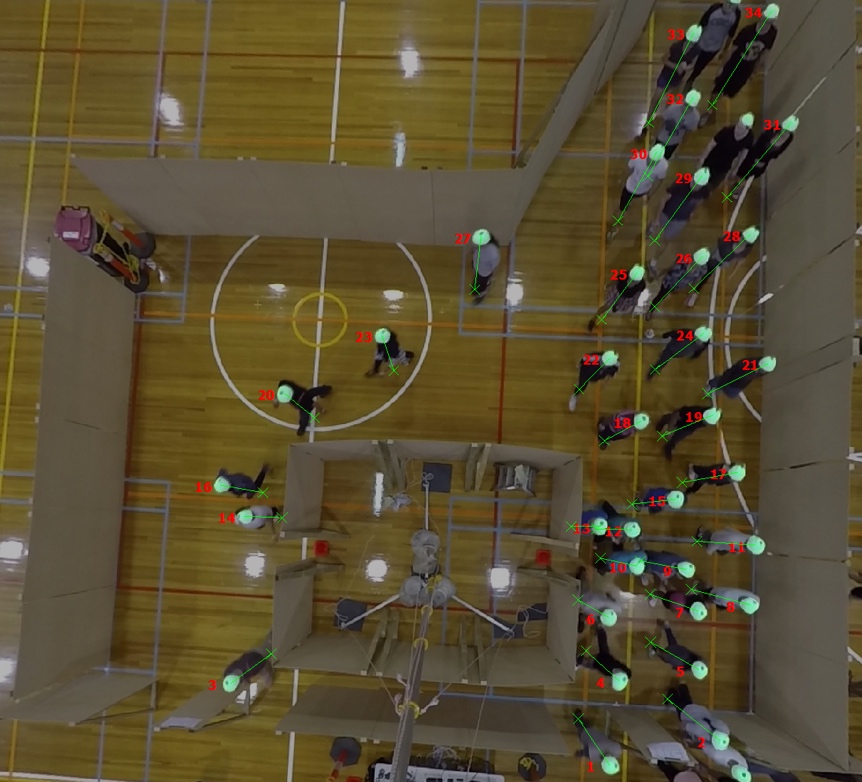


**Fig. 3.** The center of the greenish areas with an acceptable shape (size, aspect ratio) determines the position of a person. The green cross shows the projected position on the ground assuming an average size of 1.7 m.

For the calculation of the position of a person in 3D and on the floor the height of the participant or distance to the camera respectively is needed. For the assumed average height of 1.7 m the green cross in Fig. 3 shows the position on the floor for each participant in the scene. Because of the perspective distortion the maximum error for a maximum height difference to the average of 0.1 m and the maximum angel of view of 47° is 0.11 m at the image border of the maze (topmost persons in Fig. 3). Errors due to the average sizing of all persons or different perspective to the worn hats depend on the angle of view according to the moving plane and approach an error value of 0 at the pixel perpendicular to the camera.

The detected positions of the heads are tracked over time using the pyramidal iterative Lucas Kanade feature tracker. The tracker searches in regions of 150% of every persons´ head size in recursive Gaussian pyramids for similar pixel between successive frames. The results are trajectories of the movement for every single individual participating at the experiments.
